# Towards Defining Heterotic Gene Pools in Pearl Millet [*Pennisetum glaucum* (L.) R. Br.]

**DOI:** 10.3389/fpls.2017.01934

**Published:** 2018-03-02

**Authors:** A. Radhika Ramya, Lal Ahamed M, C. Tara Satyavathi, Abhishek Rathore, Pooja Katiyar, A. G. Bhasker Raj, Sushil Kumar, Rajeev Gupta, Mahesh D. Mahendrakar, Rattan S. Yadav, Rakesh K. Srivastava

**Affiliations:** ^1^Department of Genetics and Plant Breeding, Acharya N. G. Ranga Agricultural University, Guntur, India; ^2^International Crops Research Institute for the Semi-Arid Crops, Patancheru, India; ^3^All India Coordinated Research Project on Pearl Millet, Indian Council of Agricultural Research, Jodhpur, India; ^4^Centre of Excellence in Biotechnology, Anand Agricultural University, Anand, India; ^5^Institute of Biological, Environmental and Rural Sciences, Aberystwyth University, Aberystwyth, United Kingdom

**Keywords:** pearl millet, B- (maintainer) lines, R- (restorer) lines, SSR (simple sequence repeat) markers, gene diversity, PIC (polymorphism information content), heterotic groups

## Abstract

Pearl millet is a climate resilient crop and one of the most widely grown millets worldwide. Heterotic hybrid development is one of the principal breeding objectives in pearl millet. In a maiden attempt to identify heterotic groups for grain yield, a total of 343 hybrid parental [maintainer (B-) and restorer (R-)] lines were genotyped with 88 polymorphic SSR markers. The SSRs generated a total of 532 alleles with a mean value of 6.05 alleles per locus, mean gene diversity of 0.55, and an average PIC of 0.50. Out of 532 alleles, 443 (83.27%) alleles were contributed by B-lines with a mean of 5.03 alleles per locus. R-lines contributed 476 alleles (89.47%) with a mean of 5.41, while 441 (82.89%) alleles were shared commonly between B- and R-lines. The gene diversity was higher among R-lines (0.55) compared to B-lines (0.49). The unweighted neighbor-joining tree based on simple matching dissimilarity matrix obtained from SSR data clearly differentiated B- lines into 10 sub-clusters (B1 through B10), and R- lines into 11 sub-clusters (R1 through R11). A total of 99 hybrids (generated by crossing representative 9 B- and 11 R- lines) along with checks were evaluated in the hybrid trial. The 20 parents were evaluated in the line trial. Both the trials were evaluated in three environments. Based on *per se* performance, high *sca* effects and standard heterosis, F_1_s generated from crosses between representatives of groups B10R5, B3R5, B3R6, B4UD, B5R11, B2R4, and B9R9 had high specific combining ability for grain yield compared to rest of the crosses. These groups may represent putative heterotic gene pools in pearl millet.

## Introduction

Pearl millet [*Pennisetum glaucum* (L.) R. Br.] known by several names, such as bulrush millet, spiked millet, cattail millet, candle millet, *bajra*, is a climate resilient nutritious cereal (Anuradha et al., 2017). It is widely distributed across arid and semi-arid tropics of Africa and Asia and other parts of the world. It is grown in about 29 mha in more than 30 countries. The major growing areas lie in Asia (>9 mha), Africa (about 18 mha), and America (>2 mha). It is one of the most widely cultivated cereals globally, ranking after rice, wheat, maize, barley, and sorghum in terms of area planted to these crops (Khairwal et al., 2007). The out-crossing breeding biology and wider adaptive nature lead to greater levels of diversity in pearl millet (Satyavathi et al., 2013; Singh et al., 2013).

Development of high-yielding hybrids is an important breeding objective for pearl millet worldwide. The availability, assessment, and exploitation of genetic diversity help to develop new cultivars and heterotic groups which would result in hybrids with a high degree of heterosis for grain yield. The assignment of germplasm into different heterotic groups is fundamental for maximum exploitation of heterosis for hybrid development (Gurung et al., 2009). Prediction of heterosis and F_1_ performance from the parental generation could largely enhance the efficiency of breeding hybrid or synthetic cultivars by reducing the costs associated with making crosses and field evaluation for selecting heterotic crosses (Teklewold and Becker, 2005). In pearl millet, a successful heterosis breeding program rests on the development of diverse seed (A-/B- lines) and pollen/restorer (R- lines) parents with distinctly separated gene pools. Limited information is available on the classification of a large number of hybrid parental lines based on molecular marker data, while there is no information available on the identification of heterotic pools in pearl millet using genomic tools. The present study was carried out with an objective to define putative heterotic gene pools in pearl millet assisted by expressed sequence tag (EST) and genomic SSR markers.

## Materials and methods

### Genetic material and DNA extraction

The plant material used in the experiment comprised of 342 hybrid parental line of pearl millet which included 160 B- (maintainer) and 182 R- (restorer) lines along with Tift 23D_2_B_1_-P1-P5 (world reference germplasm) as control (repeated five times). These are International Crops Research Institute for the Semi-Arid Tropics (ICRISAT)-bred lines representing genetic diversity of mainly Asia and Africa. The world reference line, Tift 23D_2_B_1_-P1-P5 is a single plant selection done at ICRISAT from Tift 23D_2_B_1_ line which was bred at the Coastal Plain Experiment Station, Tifton, Georgia, USA. Tift 23D_2_B_1_-P1-P5 has recently been sequenced (Varshney et al., 2017). The list of experimental material with pedigree details are presented in Table S1. Leaf samples were collected from 15 to 20 days old seedlings and DNA isolation was carried out using high throughput DNA extraction method (Mace et al., 2003). The quantification of concentrated DNA were done on 0.8% agarose gel using Lambda DNA (New England BioLabs) as a standard. Based on the quantity of DNA, working stocks with diluted DNA were prepared at a concentration of 5 ng/μl for SSR genotyping.

### Molecular markers

Out of 124 markers used in this study, 88 SSR markers were selected for final analysis. These included 72 EST-SSRs (69 IPES and 3 ICMP) and 16 genomic SSRs. These SSRs produced clear, scorable and polymorphic profile upon PCR amplification. Number of repeats in the SSR motifs were 2, 3, 4, 5, and 6 for 30, 28, 10, 15, and 5 markers, respectively. Of the 88 SSR markers, 81 were mapped on the 7 linkage groups of pearl millet, with 14, 12, 10, 7, 6, 19, and 14 markers located on LG1, LG2, LG3, LG4, LG5, LG6, and LG7, respectively (Allouis et al., 2001; Qi et al., 2004; Senthilvel et al., 2008; Rajaram et al., 2013). The details of markers are presented in Table S2.

### PCR (polymerase chain reaction) setup

PCR reactions were carried out as per Kumar et al. (2016) using GeneAmp PCR System 9700 thermal cycler (Applied Biosystems, USA). PCR reaction mixture of 5 μl was prepared in 384 well PCR plate which comprises of 1 μl DNA template, 0.3 μl of 2 mM dNTPs, 0.12 μl of 25 mM MgCl_2_, 0.5 μl of 2 pmole/μl forward and reverse primers each, 0.5 μl of 10X PCR buffer, 0.03 μl of 0.5 U Taq DNA polymerase and the rest was double sterilized water. PCR steps comprised of 94°C for 5 min, 40 cycles for 94°C for 10 s, 54°C for 20 s, 72°C 30 s, and a final extension at 72°C for 20 min. PCR products were size separated on 1.5% agarose gel.

### SSR/microsatellite analysis

After confirmation of amplification, based on the amplicon size and forward primer label of the markers, different multiplex sets were defined to perform SSR genotyping. Each set consisted of 3–4 markers with different product sizes and labels in order to avoid ambiguity during data analysis. One microliter dye-labeled PCR products of each multiplex set were pooled and mixed with 7 μl of Hi-Di formamide, 0.15 LIZ-500 size standard (Applied Biosystems, USA) and 5 μl of distilled water. The pooled PCR amplicons were denatured for 5 min at 95°C and cooled immediately on ice. These amplicons were size separated based on the principle of capillary electrophoresis using an ABI Prism 3730 DNA analyzer (Applied Biosystems Inc.). Raw data obtained from ABI 3730 × l Genetic Analyser was subjected to analysis using the software Genemapper® version 4.0 (Applied Biosystems, USA). Based on the relative migration of internal size standard, product sizes were scored in base pairs (bp). Further analysis was done using Allelobin 2.0 program (Prasanth et al., 1997) based on repeat of SSR marker motif to get perfect allele calls.

The software package PowerMarker version 3.25 (Liu and Muse, 2005) was used to determine allele frequency, availability of data, allele number, gene diversity, heterozygosity, and polymorphic information content (PIC) from the marker data. A neighbor joining tree was constructed based on the simple matching dissimilarity matrix obtained from the marker data using DARwin 5.0.156 software (Perrier and Jacquemoud-Collet, 2006).

### Hybridization

The crossing program was undertaken at ICRISAT, Patancheru during *Summer*, 2015. Based on the genetic distance obtained from the simple matching dissimilarity matrix constructed using genotyping data, mean representatives from each group were selected for crossing. In this study, 10 B- and 12 R- lines were selected to generate 120 crosses using line × tester mating design. Later on, one B- (from B7) and one R- (from R1) line were excluded from crossing plan due to a poor plant stand. Therefore, 99 crosses made from 9 B- (lines) and 11 R- (testers) lines were taken forward. The details of the selected parental lines are given in Table 1.

**Table 1:** Details of 9 B- and 11 R- lines used in the line × tester crossing design.

**Representative entry code**	**Heterotic Group/Cluster**	**Cluster genetic distance mean**	**Representative entry number**	**Pedigree of the representative entry**	**Mean genetic distance of the representative entry**
**B- LINE CLUSTER**					
L1	Cluster B1	0.3020	74	[ICMB 97444 × (843B x 405B)-4]-1-2-B-B-B	0.2972
L2	Cluster B2	0.3565	41	[IPC 1598 × (843B × DSA 105B)]-51-3-B-B	0.3564
L3	Cluster B3	0.3707	48	(ICMB 89111 × IP 9554-9)-4-2-2	0.3739
L4	Cluster B4	0.3936	162	(ICMB 03111 × {(MC 94 S1-34-1-B × HHVBC)-16-2-1-1-1-1-B-B-5 × (MC 94 S1-34-1-B × HHVBC)-10-4-1-2-1-B-B-1-30-2-4-2-1)-7-5-4-1-1	0.3909
L5	Cluster B5	0.3146	105	[HHV-S1-24-3-B-3-2 × (ICMB 96333 × HHVBC)]-19-B-1-3-B-B-B-B	0.3268
L6	Cluster B6	0.3915	84	NC D2 S1-2-2-2-3-2-B-2	0.4157
L7	Cluster B8	0.3864	75	(ICMB 96555 × IP 10437)-2-4-2-B-6-1	0.3829
L8	Cluster B9	0.4078	25	ARD-288-1-10-1-2 (RM)-5	0.4068
L9	Cluster B10	0.4697	132	[(MC 94 S1-34-1-B × HHVBC)-10-4-3-2-2-B-B-2 × (ICMR 312 S1-1-5-3-B × HHVBC)-7-1-1-1-B-B-B]-21-B-1-2	0.4661
**R- LINE CLUSTER**					
T10	Cluster R2	0.355	222	(AIMP 92901 S1-480-1-1-1-2-B-2 × ICMR 312 S1-3-2-3-2-1-1-B-B)-B-11-1-1-B	0.3524
T11	Cluster R3	0.3529	336	[(IPC 1268 × ICMV 91059 S1-58-2-2-2-1) × AIMP 92901 S1-296-2-1-1-1-B-B]-2-2-3-2-3	0.3526
T12	Cluster R4	0.3815	201	((ICMV IS 94206 S1-15-2) × {(SRC II C3 S1-19-3-2 x HHVBC)-5-3-1})-B-13-4-2-1-1-1-1-3-2	0.3693
T13	Cluster R5	0.4139	270	MDMRRC S1-329-1	0.4110
T14	Cluster R6	0.4952	333	ICTP 8202 S1-25-1	0.4950
T15	Cluster R7	0.4745	199	JBV 3 S1-257-1-4-1-B	0.4699
T16	Cluster R8	0.5042	192	ICMS 7704-S1-127-5-1-5-1-1-3-3-2-B-B	0.5033
T17	Cluster R9	0.4565	185	[(((ICMV-IS 94206-15) × B-Lines)-B-6) × (MRC S1-156-2-1-B)]-B-13-1-3-3-2-B	0.4562
T18	Cluster R0	0.4696	172	MRC HS-219-2-1-2-B-B-B-B	0.4707
T19	Cluster R11	0.4360	174	MRC HS-130-6-1-1-B-B-B-B-B-B	0.4384
T20	Undetermined Cluster (UD)	0.4316	233	(RCB-2-S1-43-3-4 × MRC)-B-2-1-1-B-1-B	0.4324

### Evaluation of parental lines and F_1_ crosses

The parental lines and F_1_ hybrids were evaluated in two contiguous, but separate trials at three locations. In the hybrid trial, a total of 123 F_1_ hybrids along with seven checks were evaluated, while in the inbred line trial a total of 60 inbred lines (54 inbreds + 6 checks) were evaluated during rainy season, 2015 over two locations viz., ICRISAT, Patancheru and Agricultural Research Station, Vizianagaram, Acharya N. G. Ranga Agricultural University (ANGRAU); and at one location during post-rainy season, 2015 at Agricultural College Farm, Naira, ANGRAU. Both hybrid and line trials were laid out in a two-replication Alpha lattice design, where each entry was sown in 2 rows of 2 m length, spaced at 15 cm between plants and 75 cm between rows. However, only 99 hybrids and three checks viz., HHB 67 Improved, ICMH 356 and HHB 146 Improved were considered for analysis in the hybrid trial, and 20 lines (11R- lines and 9 B- lines involved in the cross combinations) were considered for the line trial.

Standard agronomic management practices were followed in each of the trials viz., basal dose of 100 kg of DAP (diammonium phosphate, containing 18% N, 46% P) was applied at the time of field preparation and 100 kg of urea (46% N) was applied as top dressing to meet the recommended dose of 64 kg of N ha^−1^ and 46 of P ha^−1^; irrigations were given soon after sowing, subsequently as and when required. Seedlings were thinned at 15 days after sowing to maintain one healthy seedling per hill at a spacing of ~15 cm. The other cultural practices like weeding, protection against insects, pests, diseases and birds were done throughout the growing period as and when required. The data on grain yield was recorded on plot basis in all the experimental trials at all locations.

### Statistical analysis

The standard/useful/economic heterosis is superiority (or inferiority) of the F_1_ hybrid in relation to the check(s). It was calculated by the formula, [(F_1_ – check)/check] × 100. Analysis of variance (ANOVA) (Panse and Sukhatme, 1985) was performed to estimate the variance components among and within B- and R-line groups. Estimates of combining ability variances and effects were obtained using line × tester method suggested by Kempthorne (1957) and detailed by Singh and Chaudhary (1985). Statistical analysis was performed using PROC MIXED model in SAS software at ICRISAT, Patancheru. In this model, block within replications were kept random, while replications and genotypes were treated as fixed.

Analysis of molecular variance (AMOVA) (Excoffier et al., 1992), was performed using the software package GenAlEx version 6.5 (Peakall and Smouse, 2012) to estimate F_ST_ index which represented the distribution of allelic diversity across multiple levels of population subdivisions. Statistical significance for F_ST_ was computed by random permutation of all the population samples. PhiST was calculated after every reshuffling step for generation of a distribution of PhiST values. “Codom-Allelic” randomization method was selected where all alleles at a single locus were randomly shuffled among individuals. Comparison of the observed F_ST_ values to the distribution of 999 permutations provided *P*-values for the B- and R- lines.

## Results

### Molecular diversity

Genetic parameters like total allele number, gene diversity, heterozygosity, and Polymorphism Information Content (PIC) are given in Table S3. The average availability [which is defined as (1 – Obs/n), where Obs is the number of observations, and n is the number of individuals sampled] of marker data for analysis was 89.0%.

#### Allele size, number, and their distribution across parental lines

The SSR markers used in the present study had allele size within a range of 108–122 bp (Xipes0066) to 409–414 bp (Xipes0205). Moreover, all the markers used in the study had shown band sizes in correspondence with the expected band sizes. The check, Tift 23D_2_B_1_-P1-P5 repeated five times along with experimental material had shown identical allele size for each of the markers indicating the robustness of the results.

A total of 532 alleles were found among 342 parental lines and check with a mean value of 6.05 alleles per locus. The number of alleles ranged from 2 (Xipes0142, Xipes0079, Xipes0026, Xipes0205, Xpsmp2235, Xpsmp2253, and Xipes0147) to 28 (Xpsmp2070) alleles per locus, followed by Xipes0233 (21), Xipes0027 (17) and Xipes0098 (16). Seventy-two out of 88 markers detected alleles within the range of 3–10 with a mean value of 5.14 alleles per locus, whereas 5 markers identified alleles within the range of 11–15 with an average of 13.20. The 72 EST-SSRs used in study resulted an average of 5.89 alleles, ranged from 2 (Xipes0142, Xipes0079, Xipes0026, Xipes0205, and Xipes0147) to 21 (Xipes0233), whereas 16 genomic SSRs identified number of alleles which varied from 2 (Xpsmp2235 and Xpsmp2253) to 28 (Xpsmp2070), with a mean value of 6.75. Out of 532 alleles, 443 (83.27%) alleles were contributed by maintainer (B-) lines with a mean of 5.03 alleles per locus, whereas restorer (R-) lines contributed 476 alleles (89.47%) with a mean of 5.41. A total of 441 (82.89%) alleles out of 532 alleles were shared commonly between B- and R- lines. All the markers were polymorphic across R-lines, while one marker Xipes0147 was found to be monomorphic among B-lines.

#### Gene diversity, heterozygosity, and polymorphism information content (PIC)

Gene diversity is defined as the probability that two randomly chosen alleles from the population are different. The average gene diversity in this study was 0.55, varied from 0.02 (Xipes0147) to 0.90 (Xpsmp2070). Out of 88 SSR markers, 60 loci showed gene diversity of equal to or more than 0.50, with a mean value of 0.66, whereas 28 markers resulted in gene diversity < 0.50 with a mean value of 0.29. The EST-SSRs and genomic SSRs recorded mean gene diversity values of 0.56 and 0.48, respectively. Based on individual analysis among B- and R- lines, pollen parents (0.55) had high average gene diversity than seed parents (0.49).

Seventy-one of the 88 SSR markers revealed heterozygosity, of which marker Xipes0226 detected maximum (0.12) heterozygotes followed by Xipes0027 (0.07) and Xipes0206 (0.07), while 21 markers could not find any heterozygotes. The mean heterozygosity was 0.02. Of 71 markers, 56 SSRs showed < 0.05 heterozygosity with an average of 0.02 and 11 SSRs had more than 0.05 heterozygosity with a mean of 0.06. The average heterozygosity was greater among R- lines (0.03) than B- lines (0.01).

The PIC ranged from 0.02 (Xipes0147) to 0.90 (Xpsmp2070) with an average of 0.50. Out of 88 markers, 50 markers showed PIC > 0.50 with a mean of 0.65, whereas 21 markers resulted in PIC values that ranged from 0.30 to 0.50, and rest of the 17 markers had PIC < 0.30. The PIC value ranged from 0.00 (Xipes0147) to 0.79 (Xipes0098) among B-lines and from 0.03 (Xpsmp2253) to 0.91 (Xpsmp2070) among R-lines with an average of 0.44 and 0.50 in B- and R- lines, respectively. The mean PIC value of EST-SSRs (0.51) was greater than that of genomic SSRs (0.45).

#### Grouping of B- and R- lines based on genetic distance

**Figure 1:**
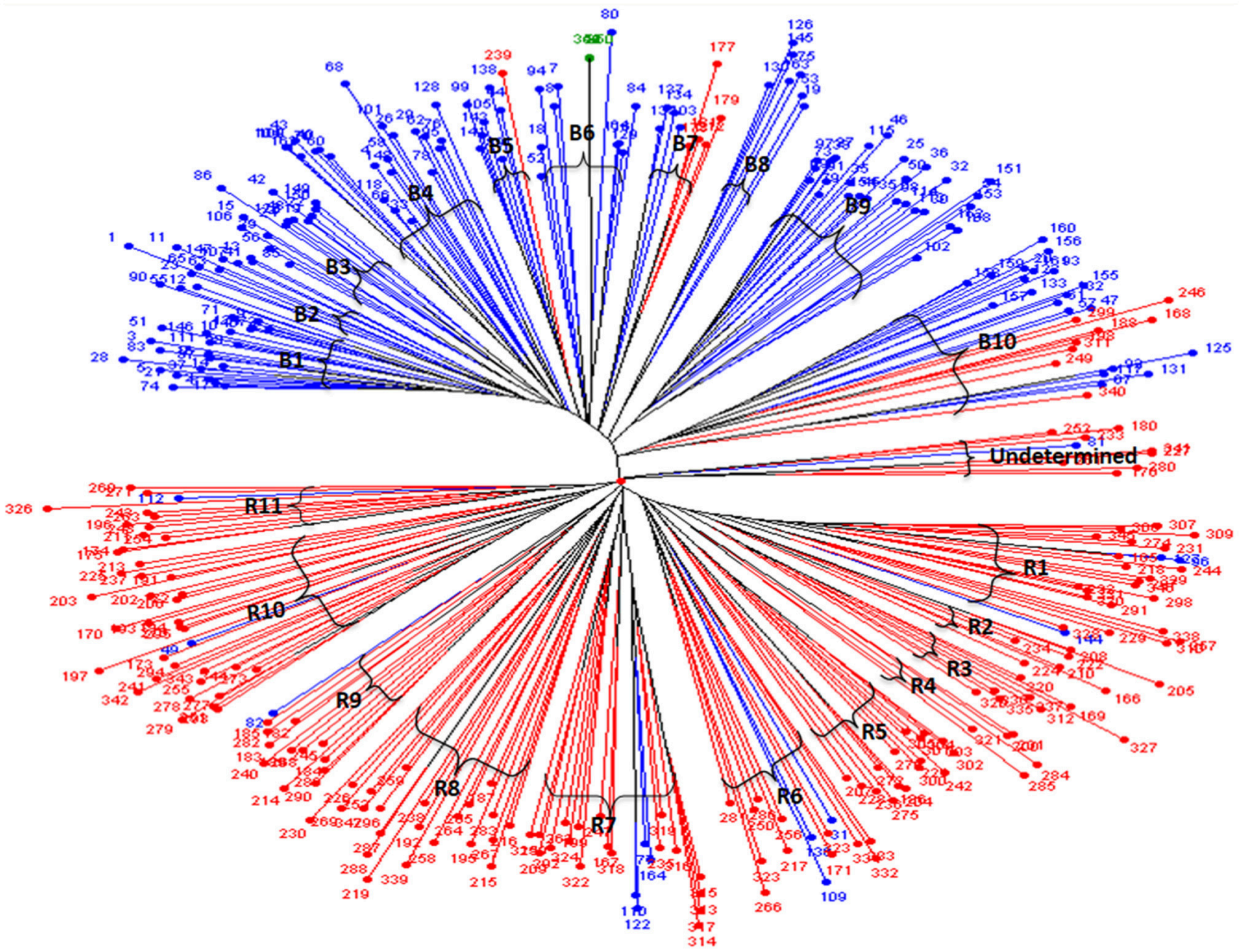
Unweighted neighbor joining tree based on the simple matching dissimilarity matrix of 88 SSR marker data for 347 hybrid parental lines. Lines in in blue are B- lines, line in green is Tift 23D_2_B_1_-P_1_-P_5_ (B-line, and the world reference genotype), lines in red are R- lines; B1 to B10 are the 10 sub-clusters of the B- lines; while R1 to R11 are the 11 sub-clusters of the R- lines.

The dendrogram generated from the cluster analysis using simple matching dissimilarity matrix obtained from SSR data depicted in Figure 1 clearly differentiated the B- lines from R- lines. The B- and R-lines were further grouped into 10 clusters and 11 clusters, respectively. Dissimilarity coefficient values for 347 lines ranged from 0.78 between line 266 of cluster R6 and 163 of cluster B8 to 0.06 between 118 and 66 in cluster B4 with an average of 0.55.

Among the clusters of B- lines, the cluster B10 was largest with 28 lines. It comprised of 20 seed parents and 8 pollen parents, while smallest cluster was B8 with 7 maintainer lines. The clusters B1, B3, B4, B6, and B9 consisted of 26, 14, 22, 15, and 27 B-lines, respectively. The remaining clusters B2 comprised of 9 B-lines, B5 grouped 7 B- and 1 R-lines and B7 had 4 B- and 5 R-lines in their clusters. Out of 10 clusters of B-lines, 6 had more than 13 lines, whereas 4 clusters possessed < 10 lines in their groups. Based on individual cluster analysis, the lines in B1, B2, B3, B4, B5, B6, B7, B8, B9, and B10 were grouped at 0.30, 0.36, 0.37, 0.39, 0.31, 0.39, 0.39, 0.39, 0.41, and 0.47 genetic dissimilarity values. The mean representative entries in each cluster on the basis of dissimilarity values were 74 (0.30), 41 (0.36), 48 (0.37), 162 (0.39), 105 (0.33), 84 (0.42), 137 (0.40), 75 (0.38), 25 (0.41), and 132 (0.47) in B1, B2, B3, B4, B5, B6, B7, B8, B9, and B10, respectively.

Amongst the clusters of R-lines, R10 was the largest with 28 lines with 27 R- lines and one B-line and R4 was the smallest cluster with 5 R-lines. The remaining clusters of R- lines were R1, R2, R3, R5, R6, R7, R8, R9, and R11 with 25 (22 R- and 3 B-lines), 7, 8, 16, 15 (12R- and 3 B-lines), 22 (18 R- and 4 B-lines), 22, 13 (12 R-lines and 1B-line), and 12 (11 R-lines and 1 B-line) lines, respectively. On the basis of individual cluster analysis, the grouping of clusters R1, R2, R3, R4, R5, R6, R7, R8, R9, R10, and R11 was done at genetic dissimilarity values of 0.41, 0.35, 0.35, 0.38, 0.41, 0.50, 0.47, 0.50, 0.46, 0.47, and 0.44, respectively. The mean representative entries in each cluster on the basis of dissimilarity values were 298 (0.40), 222 (0.35), 336 (0.35), 201 (0.37), 270 (0.41), 333 (0.50), 199 (0.47), 192 (0.50), 185 (0.46), 172 (0.47), and 174 (0.44) in R1, R2, R3, R4, R5, R6, R7, R8, R9, R10, and R11, respectively. Nine lines were included in the undetermined cluster at dissimilarity value of 0.43 and the representative entry of this cluster was 233 (0.43).

### Analysis of variance (ANOVA) and analysis of molecular variance (AMOVA)

The combined analysis of variance for grain yield of the testcross hybrids generated by crossing 9 representative B- lines with 10 representative R- lines and one representative R- line from the undetermined cluster is presented in Table 2A. The analysis of variance revealed highly significant (*P* = 0.001) differences between the clusters for different cross combinations.

**Table 2A:** Combined analysis of variance of the testcross hybrids generated on 11 representative R-lines for grain yield.

**Source of variation**	**Degree of freedom**	**Sum of squares**	**Mean sum of squares**	***F*-value**	***P*-value**
Between groups	10	1,367.2	136.72	3.21219	0.0014
Within groups	88	3,745.539	42.56294		
Total	98	5,112.739			

AMOVA was generated using genotyping data from 88 microsatellite loci for 160 B- lines and 182 R-lines. The comparison of the observed F_ST_ values to the distribution of 999 permutations provided highly significant (*P* = 0.001) differences between the 10 B- line clusters; and between 10 R- line clusters, and an undetermined group. Genetic variations among individual for B- and R- lines (96 and 94%, respectively) was significantly higher compared to within individual variance for B- and R- lines (2 and 5%, respectively) (Table 2B).

**Table 2B:** Analysis of molecular variance (AMOVA) for B- and R- line clusters.

**Source of variation**	**Degree of freedom**	**Sum of squares**	**Mean sum of squares**	**Estimated variance**	**Variance percentage (%)**	***F*-value**	***P*-value**
**B- LINES**							
Among populations	9	528.20	58.69	0.31	1	0.012	0.001
Among individuals	150	7,382.64	49.22	24.31	96		
Within individuals	160	96.50	0.60	0.60	2		
Total	319	8,007.33		25.22	100		
**R- LINES and ONE UNDETERMINED GROUP**							
Among populations	11	694.53	63.14	0.36	1	0.013	0.001
Among individuals	170	8,932.55	52.54	25.66	94		
Within individuals	182	224.50	1.23	1.23	5		
Total	363	9,851.59		27.25	100		

### Combined analysis of variance for combining ability

Analysis of variance for combining ability for different grain yield per plant based on line × tester analysis is presented in Table 3. The pooled analysis of variance showed highly significant (*P* ≤ 0.01) differences among parents, hybrids, hybrids vs. parents, parents × environment interaction, hybrid × environment interaction for grain yield per plant.

**Table 3:** Combined analysis of variance for combining ability of grain yield per plant based on pooled data of three environments.

**Source of variation**	**Numerator degree of freedom**	**Denominator degree of freedom**	***F*-value**	**Pr > *F***
**Location**	2	52.4	64.01	<0.0001
**Replication (Location)**	3	55.7	4.02	0.0117
**Treatments**	118	209	4.90	<0.0001
Hybrids	98	209	4.04	<0.0001
Hybrids-Line	8	222	3.38	0.0011
Hybrids-Tester	10	221	2.30	0.0138
Hybrids-Line × Tester	80	213	4.30	<0.0001
**Parents**	19	237	3.17	<0.0001
Parents-Line	8	242	4.65	<0.0001
Parents-Tester	10	236	2.29	0.0142
Parents-Line vs. Tester	1	242	0.54	0.4632
Hybrid vs. Parent	1	148	123.09	<0.0001
**Location × Treatment**	236	183	2.54	<0.0001
Location × Hybrids	196	192	2.64	<0.0001
Hybrids-Location × Line	16	266	2.86	0.0002
Hybrids-Location × Tester	20	267	1.46	0.096
Hybrids-Location × (Line × Tester)	160	211	2.78	<0.0001
**Location × Parents**	38	289	2.13	0.0003
Parents-Location × Line	16	284	3.57	<0.0001
Parents-Location × Tester	20	287	1.03	0.4214
Parents-Location × (Line vs. Tester)	2	216	2.02	0.1358
**Location × (Hybrid vs. Parent)**	2	110	0.54	0.5865

#### *Per se* performance of parents and hybrids, general combining ability (GCA), and specific combining ability (SCA) effects

The details on *per se* performance of parents and hybrids, general combining ability and specific combining ability effects for grain yield per plant based on pooled data of three environments are presented in Tables 4, 5. The mean values of grain yield per plant among the B- line groups varied from 15.12 (B1) to 36.91 g (B2) with a mean of 22.04 g, whereas amongst the R- line groups the range varied from 16.75 (R4) to 34.51 g (R5) with an average of 23.27 g. The mean of hybrids varied from 20.74 (B3R9) to 70.88 g (B10R5) with a mean of 33.94 g. Based on the pooled mean performance of 99 F_1_s generated from cross between a representative of each B- line group with a representative of each R- line group, cluster combination B1R3 (42.66 g), B2R4 (42.24 g), B3R5 (57.23 g), B4UD (47.73 g), B5R11 (43.88 g), B6R3 (44.25 g), B8R4 (39.87 g), B9R7 (38.26 g) and B10R5 (70.88 g) recorded high grain yield per plant than their counterparts.

**Table 4:** *Per se* performance of B- and R-lines, hybrids for grain yield per plant in line × tester analysis based on pooled data of three environments.

**Heterotic group**	***Per se***	**R2**	**R3**	**R4**	**R5**	**R6**	**R7**	**R8**	**R9**	**R10**	**R11**	**UD**
B1	15.12	33.91	42.66	29.18	32.28	39.78	32.41	29.15	29.15	38.52	28.94	41.93
B2	36.91	28.04	33.98	42.21	36.18	28.66	32.01	27.77	26.40	29.34	29.87	25.55
B3	15.96	28.99	28.62	35.02	57.23	52.00	29.98	33.03	20.74	30.92	24.56	36.81
B4	18.18	33.26	41.84	30.05	34.10	32.40	32.20	27.70	21.58	33.84	32.76	47.73
B5	16.22	25.36	32.95	34.12	36.59	36.38	38.25	34.04	25.65	28.27	43.88	31.68
B6	29.21	30.28	44.25	41.42	36.38	26.13	42.31	32.07	26.21	36.36	31.59	36.29
B8	30.51	34.08	29.85	39.87	35.79	32.66	39.61	32.28	28.27	35.11	37.86	35.42
B9	18.62	25.75	37.00	33.32	30.44	35.34	38.26	24.74	31.89	28.42	25.19	33.15
B10	17.68	38.29	40.87	41.75	70.88	40.01	32.32	33.09	29.55	36.37	34.07	24.99
*Per se*		21.39	24.05	16.75	34.51	19.49	20.54	17.64	24.30	19.68	27.77	29.81

*B1–B10, B-line cluster; R2–R11, R- line cluster; UD, undetermined group*.

**Table 5:** General combining ability (GCA) effects of parents and specific combining ability (SCA) effects of crosses for grain yield per plant based on pooled data of three environments.

**Heterotic group**	**R2**	**R3**	**R4**	**R5**	**R6**	**R7**	**R8**	**R9**	**R10**	**R11**	**UD**	**GCA**
B1	2.61	5.36	−7.56^*^	−9.23^**^	3.43	−3.27	−1.69	2.13	5.09	−3.56	6.68	0.41
B2	0.19	0.12	8.91^*^	−1.88	−4.23	−0.22	0.37	2.83	−0.65	0.82	−6.26	−3.03^**^
B3	−2.31	−8.69^*^	−1.72	15.72^**^	15.66^**^	−5.70	2.18	−6.28	−2.51	−7.93^*^	1.56	0.41
B4	2.91	5.48	−5.74	−6.46	−2.99	−2.52	−2.20	−4.49	1.36	1.22	13.43^**^	−0.54
B5	−4.96	−3.38	−1.64	−3.95	1.01	3.56	4.17	−0.39	−4.18	12.36^**^	−2.60	−0.56
B6	−1.50	6.46	4.19	−5.62	−10.70^**^	6.15	0.74	−1.30	2.44	−1.39	0.55	0.90
B8	2.52	−7.72^*^	2.87	−5.99	−3.94	3.67	1.18	0.99	1.42	5.11	−0.10	0.68
B9	−2.42	2.83	−0.29	−7.94^*^	2.13	5.71	−2.97	8.00^*^	−1.89	−4.18	1.02	−2.71^**^
B10	2.96	−0.46	0.99	25.35^**^	−0.36	−7.38^*^	−1.78	−1.49	−1.08	−2.45	−14.29^**^	4.44^**^
GCA	−3.06^**^	2.95^*^	2.38^*^	7.15^**^	1.99	1.32	−3.51^**^	−7.34^**^	−0.93	−1.86	0.90	

* Significant at P ≤ 0.05;

** *Significant at P ≤ 0.01. B1–B10, B-line cluster; R2–R11, R-line cluster; UD, undetermined group*.

The *gca* effects for grain yield per plant varied from −3.03^**^ (B2) to 4.44^**^ (B10) among B- line groups and among R- line groups the range varied from −7.34^**^ (R9) to 7.15^**^ (R5). Only one B- line, B10 (4.44^**^) and three R- lines, R5 (7.15^**^), R3 (2.95^*^) and R4 (2.38^*^) exhibited positive and significant *gca* effects whereas two B- lines, B2 (−3.03^**^) and B9 (−2.71^**^) and three R-lines, R9 (−7.34^**^), R8 (−3.51^**^), and R2 (−3.06^**^) showed negative and significant *gca* effects.

Among 99 hybrids, *sca* effects varied from −14.29^**^ (B10UD) to 25.35^**^ (B10R5). Nine and seven hybrids possessed significant negative and positive *sca* effects, respectively. Of seven hybrids (mean ranged from 31.89 to 70.88 g) with specific combining ability in a desirable direction, three crosses had at least one best general combiner for this trait as their parent. The cross combination, B10R5 (25.35^**^) with H^+^ *gca* x H^+^ *gca* parental combination, showed highest significant *sca* effect followed by B3R5 (15.72^**^) (L^+^ *gca* x H^+^ *gca*), B3R6 (15.66^**^) (L^+^ *gca* x L^+^ *gca*), B4UD (13.43^**^) (L^−^ *gca* x L^+^ *gca*), B5R11 (12.36^**^) (L^−^ *gca* x L^−^ *gca*), B2R4 (8.91^*^) (H^−^ *gca* x H^+^ *gca*), and B9R9 (8.00^*^) (H^−^ *gca* x H^−^ *gca*).

#### Standard heterosis

The estimates of standard heterosis for yield over standard checks (HHB 67 Improved, ICMH 356 and HHB 146 Improved) of 99 F_1_s is presented in the Table 6. The range of standard heterosis over check HHB 67 Improved ranged from −48.86^*^ (B3R9) to 74.12^**^ (B10R5), for the check, ICMH 356, it ranged from −49.50^*^ (B3R9) to 71.93^**^ (B10R5), and for the check, HHB 146 Improved, the standard heterosis ranged from −42.67 (B3R9) to 95.21^**^ (B10R5). Positive heterosis is a desirable feature for this trait. Two of 99 hybrids, B10R5 (95.21^**^), and B3R5 (57.10^*^) recorded significant positive standard heterosis over the superior check, HHB 146 Improved.

**Table 6:** Standard heterosis over three checks for grain yield per plant using data of three environments.

**Heterotic group**	**Standard checks**	**R2**	**R3**	**R4**	**R5**	**R6**	**R7**	**R8**	**R9**	**R10**	**R11**	**UD**
B1	HHB 67 Improved	−16.96	4.08	−28.79	−20.66	−3.11	−20.91	−28.85	−28.76	−5.30	−29.08	3.01
	ICMH 356	−18.01	2.77	−29.68	−21.65	−4.33	−21.91	−29.74	−29.66	−6.49	−29.97	1.72
	HHB 146 Improved	−6.91	16.68	−20.16	−11.05	8.62	−11.34	−20.23	−20.14	6.17	−20.49	15.49
B2	HHB 67 Improved	−31.76	−16.43	2.06	−11.32	−29.78	−21.65	−31.17	−35.36	−28.24	−26.52	−36.48
	ICMH 356	−32.61	−17.48	0.77	−12.44	−30.66	−22.63	−32.04	−36.17	−29.15	−27.44	−37.28
	HHB 146	−23.49	−6.31	14.41	−0.59	−21.28	−12.16	−22.84	−27.53	−19.56	−17.62	−28.79
B3	HHB 67 Improved	−28.55	−30.49	−15.09	40.13	27.03	−26.89	−18.97	−48.86^*^	−25.60	−40.00	−10.56
	ICMH 356	−29.45	−31.37	−16.15	38.37	25.43	−27.81	−19.99	−49.50^*^	−26.54	−40.75	−11.68
	HHB 146 Improved	−19.90	−22.08	−4.80	57.10^*^	42.41	−18.04	−9.15	−42.67	−16.60	−32.73	0.28
B4	HHB 67 Improved	−17.97	2.77	−27.23	−16.38	−20.48	−20.98	−31.87	−47.10^*^	−17.08	−19.51	16.74
	ICMH 356	−19.00	1.48	−28.14	−17.43	−21.48	−21.97	−32.73	−47.76^*^	−18.12	−20.53	15.28
	HHB 146 Improved	−8.04	15.21	−18.41	−6.26	−10.85	−11.41	−23.62	−40.69	−7.04	−9.77	30.88
B5	HHB 67 Improved	−38.25	−19.56	−16.82	−10.44	−10.84	−6.65	−16.50	−36.90	−30.88	7.09	−22.15
	ICMH 356	−39.03	−20.57	−17.86	−11.56	−11.96	−7.83	−17.55	−37.69	−31.75	5.75	−23.13
	HHB 146 Improved	−30.77	−9.82	−6.75	0.41	−0.05	4.65	−6.38	−29.25	−22.51	20.06	−12.72
B6	HHB 67 Improved	−25.48	7.96	1.34	−11.22	−36.31	2.26	−21.16	−35.31	−10.98	−22.44	−11.20
	ICMH 356	−26.42	6.60	0.06	−12.34	−37.11	0.97	−22.15	−36.12	−12.10	−23.41	−12.32
	HHB 146 Improved	−16.46	21.03	13.61	−0.47	−28.60	14.64	−11.61	−27.48	−0.20	−13.05	−0.45
B8	HHB 67 Improved	−16.53	−27.27	−2.72	−12.08	−21.67	−2.96	−20.66	−30.66	−14.24	−7.46	−13.08
	ICMH 356	−17.58	−28.18	−3.94	−13.18	−22.65	−4.18	−21.66	−31.53	−15.32	−8.62	−14.17
	HHB 146 Improved	−6.43	−18.46	9.06	−1.43	−12.18	8.79	−11.05	−22.26	−3.86	3.74	−2.55
B9	HHB 67 Improved	−37.03	−9.26	−18.49	−25.54	−13.84	−5.85	−39.80	−21.81	−30.28	−38.06	−18.71
	ICMH 356	−37.82	−10.40	−19.52	−26.47	−14.92	−7.03	−40.55	−22.79	−31.16	−38.84	−19.73
	HHB 146 Improved	−29.41	1.73	−8.62	−16.52	−3.41	5.55	−32.51	−12.34	−21.84	−30.56	−8.87
B10	HHB 67 Improved	−6.19	0.27	2.26	74.12^**^	−3.02	−21.13	−18.88	−27.02	−10.74	−17.05	−39.72
	ICMH 356	−7.37	−0.99	0.97	71.93^**^	−4.24	−22.12	−19.90	−27.94	−11.86	−18.09	−40.48
	HHB 146 Improved	5.16	12.41	14.64	95.21^**^	8.73	−11.58	−9.06	−18.18	0.07	−7.00	−32.42

* Significant at P ≤ 0.05;

** *Significant at P ≤ 0.01. UD, undetermined group*.

## Discussion

Heterosis has been an area of intense research in many cross-pollinated and a few self- and often cross-pollinated crops for over a century. It has been defined as the superior (or inferior) performance of the F_1_ hybrid relative to the mid-parent value (mid-parent/average heterosis), or to the better parent (better-parent heterosis or heterobeltiosis), or over a suitable check cultivar (standard heterosis).

Identification of heterotic grouping is an important exercise in crop species where hybrids are prevalent. In pearl millet so far no information is available on heterotic gene pools using genomic tools. In this first report, we used SSR-based groupings to generate information on heterotic groups in pearl millet. We used the world reference genotype Tift23 D_2_B_1_-P1-P5 as a check in our study. The identical allele size of the check for each marker indicated the accuracy of protocol and reproducibility of the allelic data for the set of markers used in this study. The number of alleles obtained in the present investigation was higher than the earlier reports in pearl millet (Chandra-Shekara et al., 2007; Chakauya and Tongoona, 2008; Satyavathi et al., 2013; Singh et al., 2013; Sumanth et al., 2013; Kapadia et al., 2016). On the other hand, higher number of alleles per locus were detected than present study in pearl millet (Mariac et al., 2006; Kapila et al., 2008; Stich et al., 2010; Nepolean et al., 2012). The variation in allele number from one study to other might be due to type of material/sample (less or more diverse), sample size, type, and number of markers and repeat motifs of markers used in the investigation (Yang et al., 2010). A maximum number of alleles was identified by the marker Xpsmp2070, which was in agreement with the finding of Nepolean et al. (2012).

Markers with high gene diversity resulted in more number of alleles among the lines used in the study. The average gene diversity among germplasm of pearl millet was lower than earlier reports of Mariac et al. (2006) in wild sample, Stich et al. (2010), Nepolean et al. (2012). The lower average gene diversity in the present study than earlier findings might be due to type, size of sample, type, and number of markers. For instance, Mariac et al. (2006) detected gene diversity of 0.49 and 0.67 among cultivated and wild samples of pearl millet respectively. The gene diversity was found to be high among R-lines than B-lines as in Nepolean et al. (2012).

The greater heterozygosity observed in R-lines than B-lines was in correspondence with the findings of Nepolean et al. (2012). Even though pearl millet is a highly cross pollinated crop, the amount of heterozygosity observed among inbred lines was very less, which could be due to homogeneous and homozygous nature of inbreds obtained from several generations of directional selections and selfings. The small amount of heterozygosity found in experimental materials may be due to high mutational rate and mutational bias at the SSR loci (Udupa and Baum, 2001).

PIC is the best indicator for identification of most informative markers. A total of 50 markers were found to be highly informative with PIC ≥ 0.50, and can be used for discrimination of genotypes. The average PIC value in present study was higher than that reported in pearl millet inbred lines (Singh et al., 2013; Sumanth et al., 2013; Kapadia et al., 2016), but lower than that reported in pearl millet (Nepolean et al., 2012; Satyavathi et al., 2013). The average PIC of B-lines was lower than R-lines was in accordance with the study of Nepolean et al. (2012). Detection of high gene diversity and PIC among EST-SSRs than genomic SSRs revealed that EST-SSR markers had high discriminative power than genomic SSR markers, which is supported by Ramu et al. (2013). It might also be due to higher directional selection for a different set of traits in B- and R- lines, resulting in more genetic diversity in the intra-genomic regions over inter-genomic regions. Detection of a maximum number of alleles, highest gene diversity and high PIC by Xpsmp2070 among maintainer and restorer lines of pearl millet was in corroboration with the finding of Nepolean et al. (2012).

The clear differentiation of B- lines from R-lines with some intrusions was in correspondence with the finding of Nepolean et al. (2012). Based on genetic dissimilarity values, grouping of inbred lines was done at an average genetic distance of 0.55 indicated the presence of a moderate level of genetic variation among the lines used in the study. Likewise, many findings on grouping of germplasm based on marker genetic distance were reported in pearl millet by Stich et al. (2010), Nepolean et al. (2012), Satyavathi et al. (2013), Singh et al. (2013), Sumanth et al. (2013), and Kapadia et al. (2016).

The lines with similar a pedigree in their parentage are grouped together in the same cluster with minor deviations indicated the precision of marker-based genetic distance in grouping of the diverse parental lines. For example, among B- line clusters, in B1, 21 lines had 843B as a common parent in their pedigree, while remaining lines possessed mixed parentage. The check Tift 23D_2_B_1_-P1-P5 which was repeated five times in the experiment grouped in B6, indicating the accuracy of the protocol adopted and reproducibility of the analyzed data. In B10, 14 lines had (MC 94 S1-34-1-B × HHVBC) in their parentage. In addition, B10 comprised of eight R-lines, of which six had [((MC 94 S1-34-1-B × HHVBC)-16-2-1) × (IP 19626-4-2-3)] in their pedigrees.

Likewise, amongst the R- line clusters, one B- line grouped with R-lines in R1 cluster, which might be due to its common parentage with most of the lines in the corresponding cluster. The lines with ICMR 312 in their parentage grouped in R2, while lines with AIMP 92901 clustered in R3. The cluster R5 is dominated by lines derived from crosses involving ICMR 312 S1-3-2-1-2-4 in their parentage. Majority of the lines possessed (SRC II C3 S1-19-3-2 × HHVBC) commonly in their parentages in R7, where B- lines were grouped in this cluster which could be due to involvement of cross combination (SRC II C3 S1-19-3-2 × HHVBC) in their parentages. In the cluster R10, out of 28, 16 lines possessed MRC series in their pedigree. A similar result of coincidence of clustering patterns based on marker distance with pedigree data was given by Satyavathi et al. (2013).

The presence of significant phenotypic differences for grain yield between the 11 R- line groups involved in a total of 99 crosses, and significant molecular differences among B- and R- line clusters (Tables 2A,B) suggested the existence of sufficient phenotypic and genetic variation in the experimental material for heterotic gene pool formation exercise.

Based on the *gca* effects, one B-line 132 (B10) and three R- lines 336 (R3), 201 (R4), and 270 (R5) were found as good general combiners for the trait, grain yield per plant. Therefore the lines of groups (represented by mean representative entries) with trait of interest can be utilized in breeding program straightaway as parents for production of hybrids by crossing with other divergent lines or may be used in the line development programs.

Seven cross combinations, B10R5, B3R5, B3R6, B4UD, B5R11, B2R4, and B9R9 with high specific combining ability effects in desirable direction were obtained from the parental combinations of (H^+^ *gca* × H^+^ *gca*), (L^+^ *gca* × H^+^ *gca*), (L^+^ *gca* × L^+^ *gca*), (L^−^ *gca* × L^+^ *gca*), (L^−^ *gca* × L^−^ *gca*), (H^−^ *gca* × H^+^ *gca*), (H^−^ *gca* × H^−^ *gca*), respectively. The cross between two high general combiners revealed additive and additive × additive genetic components of variance. The cross between high × low general combiners that resulted in superior cross combination might be due to complimentary action arising out of both additive and non-additive genetic components. The superiority of the crosses having low *gca* parents may be due to high nicking ability and high *sca* effects for the parents. It will, therefore, be rewarding to design hybrid breeding programs which precisely estimate not just *gca* effects, but also *sca* effects of the hybrid parental lines by making factorial crosses with the right set of testers. Also, since every B- and R- line cluster was different and distinct in terms of genetic distance, the presence of heterotic combinations in just a few groups over others, suggests a more complex interaction of genetic distance with *sca* effects. This result is similar to Pucher et al. (2016) who reported statistically non-significant differences in the grain yield between the inter- and intra-country crosses in pearl millet.

Based on overall performance (*per se* performance, high *sca* effects and standard heterosis over superior check), the best heterotic cross combinations identified for grain yield per plant were obtained from F_1_s generated from mean representatives of groups B3 and B10 with representative of group R5. Other high yielding cross combinations were obtained between groups B1 and R3, B2 and R4, B3 and R5, B4 and undetermined cluster, B5 and 11R, B6 and R3, B8 and R4, B9 and R7 and B10 and R5. This clearly suggests that the crosses between the given B × R combinations from the specific clusters resulted in higher grain yield compared to the other groups. These may be due to a high degree of gene complementation and dispersion of favorable alleles between the groups for the manifestation of a higher degree of heterosis. These groups may represent putative heterotic gene pools in pearl millet.

## Conclusion

The current study is a step closer toward defining heterotic gene pools in pearl millet. A relatively large number of B- and R- lines were grouped using SSR-assisted genetic distances. Their representative testcross hybrids were evaluated in three environments in this study, which shed light on the existence of putative heterotic gene pools in B- and R- lines for the first time. However, these heterotic groups need to be further refined and broadened by selecting more appropriate set of testers for maximizing combining ability, and by evaluating the testcross hybrids in more number of representative environments.

Apart from further study on the genetic aspects, it might be interesting to integrate epigenomics, metabolomics, proteomics, and systems biology approaches for gaining better insights into the heterotic gene pools of pearl millet.

## Author contributions

RS planned and coordinated this study. ARR, PK, AGBR generated lab and field data. AR helped in data analysis. RS, CS, RG, RY, LA provided technical guidance during the conduct of research work. RS, ARR, RG, CS, SK, RY, MM drafted the manuscript. RS critically revised the paper for final publication.

### Conflict of interest statement

The authors declare that the research was conducted in the absence of any commercial or financial relationships that could be construed as a potential conflict of interest.
