# Supplementary material for: Towards Defining Heterotic Gene Pools in Pearl Millet [Pennisetum glaucum (L.) R. Br.]
Source: Front Plant Sci. 2018 Mar 2;8:1934. doi: 10.3389/fpls.2017.01934 (PMC5841052; doi:10.3389/fpls.2017.01934)
Supplement: Supplementary file 1 [file Table1.DOCX]

**Table S1: The list of experimental material with pedigree details**

| **S No** | **Name** | **Accession identity** |
| --- | --- | --- |
| 1 | Togo-11-5-2 selection | 1 |
| 2 | [(81B x SRL 53-1) x 843B]-30-2-B | 2 |
| 3 | [843B x (Boudama-481 x Ankoutess-2)-4]-2-B | 3 |
| 4 | [843B x (J 1623 x 3/4 EB-96-1-10)]-5-2 | 4 |
| 5 | (81B x 843B)-11-1-1-B | 5 |
| 6 | (843B x 81B)-30-1-1 | 6 |
| 7 | (843B x ICMPS 1500-7-4-1-6)-23-1-B-1-4 | 7 |
| 8 | [843B x (ICMPS 500-4-4-3 x ICMPS 1800-3-1-2-C3-4)]-7-1-3 | 8 |
| 9 | (843B x ICMPS 900-9-3-2-2)-41-2-6-2-2 | 9 |
| 10 | [(81B x SRL 53-1) x 843B]-30-1-1 | 10 |
| 11 | (26B x 834B)-11-2-B-B | 11 |
| 12 | (843B x Togo plot# 26-1)-27-B | 12 |
| 13 | (843B x 405B)-4-B | 13 |
| 14 | [{843B x (GNS x SS-48-40-4)-1-9-8}-30-B-B-1 x {843B x (843B x 700651)-11-1-2-B}]-39-B | 14 |
| 15 | [{843B x (GNS x SS-48-40-4)-29-7-4-B} x (843B x ICMPES 29)-23-2-3]-16-B | 15 |
| 16 | (81-1164 DB/85-1856 LR-16-B x 843DMR1)-14-6-3 | 16 |
| 17 | DMR1 S2-96-2-3-4 | 17 |
| 18 | (843B x 81B)-58-1-1-1 | 18 |
| 19 | [{26B x (81B x SRL 50-1)}-1-1-2 x 852B]-69-1-1 | 19 |
| 20 | [{{843B x (843B x 700651)-11-1-2-B} x 1163B} x (ICMB 89111 x ICMB 88005)]-5-2-2 | 20 |
| 21 | (SPF3/S91-327 x SPF3/S91-5)-6-2-3 | 21 |
| 22 | HTBC HS-48-B-1-1-1-1 | 22 |
| 23 | [(ICMB 88006 x ICMB 88005) x (ICMB 89111 x ICMB 88004)]-28-2-B | 23 |
| 24 | ICMB 97333 | 24 |
| 25 | ARD-288-1-10-1-2 (RM)-5 | 25 |
| 26 | (843B x ICTP 8202-161-5)-19-2-1-B-B-2 | 26 |
| 27 | (BSECBPT/91-40 x SPF3/S91-529)-12-1-1-5 | 27 |
| 28 | {[(81B x SRL 53-1) x 843B]-3-5-2 x (843B x 834B)-25-B-B-1}-84-5-B-B | 28 |
| 29 | (ICMB 89111 x IPC 1466)-21-1-3-6-B-5 | 29 |
| 30 | [(F4FC 1498-1-1-3 x J 104)-11-2-1-1]-7-3-1-B | 30 |
| 31 | (843B x ICTP 8202-161-5)-17-1-2-B-1 | 31 |
| 32 | (BSECBPT/91-40 x SPF3/S91-94)-3-1-1-2 | 32 |
| 33 | [(84-3869/85-4414)-8-B x 843DMR1]-1-3-2-B | 33 |
| 34 | (BSECBPT/91-38 x SPF3/S91-529)-10-1-7 | 34 |
| 35 | (BSECBPT/91-40 x SPF3/S91-3)-1-2-2-4 | 35 |
| 36 | {[(81B x SRL 53-1) x 843B]-3-5-2 x (843B x 834B)-25-B-B-1}-84-6-B-B | 36 |
| 37 | (BSECBPT/91-40 x SPF3/S91-529)-12-1-1-7-1 | 37 |
| 38 | (SPF3/S91-544 x SPF3/S91-5)-5-1-2-1 | 38 |
| 39 | [(ICMB 89111 x ICMB 88002) x {(81B x SRL 53-1) x 843B}-3+]-31 | 39 |
| 40 | [IPC 1598 x (843B x DSA 105B)]-51-3-B-B | 40 |
| 41 | (ICMB 89111 x 863B)-65-8-B-B | 41 |
| 42 | [(ICMB 89111 x ICMB 88004) x (ICMB 88006 x IP 9402)-2-1-1-4]-102-B-1 | 42 |
| 43 | {[(81B x SRL-53-1) x 843B]-3-5-3 x [(843B x 111B)-10-1-2-2]}-226-B-2-B-B-B | 43 |
| 44 | (BSECBPT/91-39 x SPF3/S91-116)-15-2-1-2 | 44 |
| 45 | (BSECBPT/91-38 x SPF3/S91-529)-2-1-B-2 | 45 |
| 46 | ICMV 87901-175-2-3-2-B-1 | 46 |
| 47 | (ICMB 89111 x IP 9554-9)-4-2-2 | 47 |
| 48 | 690-93B | 48 |
| 49 | (SPF3/S91-933 x SPF3/S91-3)-8-1-1-B | 49 |
| 50 | (843B x EEBC S1-407)-12-3-B | 50 |
| 51 | (ICMB 96111 x 4026-1-6-B)-4-2-1-1 | 51 |
| 52 | (SRC II C3 S1-19-3-2 x HHVBC)-17-3-1-3 | 52 |
| 53 | [(843B x ICTP 8202-161-5)-20-3-B-B-3 x B-bulk]-2-B-1-3 | 53 |
| 54 | (EBC-S1-40-2-2-1 x B-bulk)-25-B-B | 54 |
| 55 | [{(ICMB 89111 x ICMB 88004) x (ICMB 88006x ICMB 88005)-2-1-1-4}-101 x B-bulk]-5-B-B | 55 |
| 56 | (ICMR 312 S1-8-3-3-B x HHVBC)-9-4-1-1 | 56 |
| 57 | [(BSECBPT/91-39 x SPF3/S91-116)-15-2-1-4-4 x B-bulk]-1-B-4-1 | 57 |
| 58 | (HTBLN/95-98 x ICMB 89111)-1-B-B-1 | 58 |
| 59 | (9035/S92-B-3 x B-line bulk)-5-B-B | 59 |
| 60 | [{{{843B x (843B x 700651)-11-1-2-B} x 1163B} x ICMB 89111x ICMB 88005)}-27+ x B-bulk]-3-B-B-10 | 60 |
| 61 | (ICMB 96333 x HHVBC-2-D2-HS-259-2)-4-B | 61 |
| 62 | (ICMB 96111 x 4038-4-2-B)-2-1-5-4 | 62 |
| 63 | (D2BLN/95-262 x EEBC C1-3)-12-B-1-B-B-4 | 63 |
| 64 | [ICMB 97444 x (D2BLN/95-98 x EEBC C1-1)-7-B-B]-34-2-4-B-B | 64 |
| 65 | [(MC 94 S1-81-1-B x HHVBC)-4-4-1 x (MC 94 S1-81-1-B x HHVBC)-4-2-4]-7-1-1-B | 65 |
| 66 | [ARD-288-1-10-1-2 (RM)-3 x B-bulk]-14-B-1-1 | 66 |
| 67 | {ICMB 99555 x [(78-7088/3/SER3 AD//B282/(3/4 EB) x PBLN/S95-359)-19-5-B-B]}-13-2-B-B-B-B | 67 |
| 68 | [HTBLN/95-98 x (SPF3/S91-544 x SPF3/S91-5)-5-1-2]-3-B-B-1-B-1-B | 68 |
| 69 | (HTBC 48-B-1-1-1-5 x B-bulk)-25-1-B-B | 69 |
| 70 | (DMR 133 x HTBC 48-B-1-1-1-5)-4 | 70 |
| 71 | [78-7088/3/SER3 AD//B282/(3/4)EB x PBLN/S95-359]-19-2-B-1-B-B-3 | 71 |
| 72 | [ICMB 97444 x (843B x 405B)-4]-1-2-B-B-B | 72 |
| 73 | (ICMB 96555 x IP 10437)-2-4-2-B-6-1 | 73 |
| 74 | [(D2BLN/95-93 x SPF1/K95-3213-20)-10 x (91777B x HHVBC)]-7-B-1-B-B-4-B-1 | 74 |
| 75 | (SRC II C3 S1-19-3-2 x HHVBC)-27-2-2-1-2-2 | 75 |
| 76 | [ICMB 99555 x {78-7088/3/SER3 AD//B282/(3/4)EB x PBLN/S95-359}-10-2-B-2]-19-2-B-B-B-B | 76 |
| 77 | [ICMB 99555 x {78-7088/3/SER3 AD//B282/(3/4)EB x PBLN/S95-359}-10-2-B-2]-18-3-B-B-B-B | 77 |
| 78 | [(SRC II C3 S1-103-1-1 x HHVBC)-20 x (81B x ICMP 451)-5-4-2-3]-5-2-1-B-B-3-B | 78 |
| 79 | EEDBC S1-465-3-2-5-5 | 79 |
| 80 | [(ICMB 95111 x 9035/S92-B-3)-17-1-B-B-B-B | 80 |
| 81 | (ICMB 95444 x ICMB 94555)-18-3-B-4-2 | 81 |
| 82 | NC D2 S1-2-2-2-3-2-B-2 | 82 |
| 83 | [ICMB 97444 x (843B x 405B)-4]-1 -2-B-B-B-B | 83 |
| 84 | (ICMR 312 S1-4-3-3-B x HHVBC)-3-2-1-2-2-2 | 84 |
| 85 | (ICMB 95444 x ICMB 94555)-18-3-B-4-2 | 85 |
| 86 | [ICMB 97444 x (843B x EEBC S1-407)-12-4-B-B]-6-1-B-B-B-B | 86 |
| 87 | (ICMB 01666 x ICMB 01222)-27-3-B-3 | 87 |
| 88 | [EBC-GEN-S1-40-2-2-1 x B-bulk]-19-B-B-2-B-B-3 | 88 |
| 89 | [ICMB 99555 x {78-7088/3/SER3 AD//B282/(3/4)EB x PBLN/S95-359}-10-2-B-2]-8-1-B-2-B-B | 89 |
| 90 | [(MC 94 S1-81-1-B x HHVBC)-4-4-1 x (MC 94 S1-81-1-B x HHVBC)-4-2-4]-10-3-1-B-1 | 90 |
| 91 | (MC 94 S1-34-1-B x HHVBC)-16-2-1-4-2-B | 91 |
| 92 | (D2BLN/95-103 x EEBC C1-3)-6-B-4-B-2-1 | 92 |
| 93 | [(D2BLN/95-93 x SPF1/K95-3213-20)-10 x (91777B x HHVBC)]-7-B-1-B-B-2-B | 93 |
| 94 | MC 94 C2-S1-3-1-3-3-1-1-2-B-B | 94 |
| 95 | [HHV-S1-24-3-B-3-2 x (ICMB 91777 x HHVBC)]-5-B-1-1-B-B-B | 95 |
| 96 | IC-CZBC-C0-1-6-3-3-B | 96 |
| 97 | (ICMB 93333 x ICMB 01222)-11-1-B-7-2 | 97 |
| 98 | (ICMB 01666 x ICMB 01222)-49-1-2-4-B | 98 |
| 99 | (ICMB 99555 x ICMB 00555)-5-4-3-B-B-3 | 99 |
| 100 | [(843B x ICTP 8202-161-5)-20-3-B-B-3 x B-bulk]-2-B-2-B-1-3 | 100 |
| 101 | [ICMB 99555 x {78-7088/3/SER3 AD//B282/(3/4)EB x PBLN/S95-359}-10-2-B-2]-12-3-B-B-B-B | 101 |
| 102 | (ICMB 01666 x ICMB 01222)-29-2-B-5-1-2 | 102 |
| 103 | [HHV-S1-24-3-B-3-2 x (ICMB 96333 x HHVBC)]-19-B-1-3-B-B-B-B | 103 |
| 104 | (ICMB 97444 x 9035/S92-B-3)-7-1-1-B-B-3-B-B | 104 |
| 105 | [ICMB 96111 x 4017-2-1-B)-7-2-3 x (SRC II C3 S1-19-3-2 x HHVBC)-17-3]-1-3-4-1-1-B | 105 |
| 106 | [ICMB 95111 x (D2BLN/95-107 x EEBC C1-1)-6-B]-24-4-1-B-B-B-B-11-1] x B-bulk (3981-3989/S06 G1)}-2-3-1-B | 106 |
| 107 | NC D2 BC7F4-12-1-2-3-1-4-3-B-B | 107 |
| 108 | (SRC II C3 S1-19-3-2 x HHVBC)-12-4-1-3-2-1-B-2-B-4-B-B | 108 |
| 109 | [(ICMB 95555 x ICMB 94333)-8-2-1-B-B-9-1 x B-bulk (3981-4011/S06 G1)]-2-3-4-B | 109 |
| 110 | [EEDBC S1-452-3-1-2-3-B-B-B-1 x B-bulk (3981-3989/S06 G1)]-4-2-4-B | 110 |
| 111 | {[(843B x ICTP 8202-161-5)-20-3-B-B-3 x B-bulk]-2-B-1-2-2-B-B-B-11-1 x B-bulk (3981-4011/S06 G1)}-3-2-4-4 | 111 |
| 112 | (ICMB 04888 x ICMB 00444)-7-1-3-2 | 112 |
| 113 | (HHVDBC HS-246-1-2-1-2 x ICMB 98444)-4-3-1-2 | 113 |
| 114 | [(81B x 4017-6-1-1)-3-1-3-3-1-1-B-B x ICMB 04111]-89-2-3-4 | 114 |
| 115 | {(MC 94 S1-81-1-B x HHVBC)-4-4-1 x (MC 94 S1-81-1-B x HHVBC)-4-2-4-10-3-1--B-B-B x ICMB 02777}-24-3-2 | 115 |
| 116 | [ICMB 97444 x (D2BLN/95-98 x EEBC C1-1)-7-B-B]-34-2-4-B-B-B-5-B-B | 116 |
| 117 | (ICMB 89111 x 863B)-58-2-B-2-B-B | 117 |
| 118 | [(DMR 133 x HTBC 48-B-1-1-1-5)-19-2-B-B x ICMB 04111]-145-6-2-4 | 118 |
| 119 | (SRC II C3 S1-19-3-2 x HHVBC)-12-4-1-3-2-1-B-5-B-3-B-B | 119 |
| 120 | {(MC 94 S1-34-1-B x HHVBC)-16-2-1-1-1-1-B-B-5 x (MC 94 S1-34-1-B x HHVBC)-10-4-1-2-1-B-B-1-30-2-4-3-3 | 120 |
| 121 | BxB early maintainer (Iso F2 bulk)-85-3-2 x MRC HS-179-1-1-2-B-B-B-B-B | 121 |
| 122 | {(MC 94 S1-81-1-B x HHVBC)-4-4-1 x (MC 94 S1-81-1-B x HHVBC)-4-2-4-10-3-1-B-B-B-2 | 122 |
| 123 | [(ICMB 96555 x IP 10437)-3-4-1-2-3x{(96555B x LaGrap C2 S1-32-1)-10}xIP 14758-2-1]-2-B-1-3-1-3 | 123 |
| 124 | (ICMB 96555 x IP 10437)-3-4-1-2-2-1-B-3-B-2-B-B | 124 |
| 125 | EBC (G-5)-56-1-B | 125 |
| 126 | [(81B x 4017-6-1-1)-3-1-3-3-1-1-B-B x ICMB 04111]-86-4-4 | 126 |
| 127 | [{(ICMB 96555 x IP 10437)-9-B-B-B-B-B-B x IP 14758-2-2}-19-1-B x (ICMB 96555 x IP 10437)-3-4-1-2-2-1-B-2-B-3]-3-3-2-1-1 | 127 |
| 128 | {[(MC 94 S1-81-1-B x HHVBC)-4-4-1 x (MC 94 S1-81-1-B x HHVBC)-4-2-4-7-1-1-B-2] x (ICMR 312 S1-1-5-2-B x HHVBC)-10-2-1-2}-30-2-4-1 | 128 |
| 129 | [(MC 94 S1-34-1-B x HHVBC)-10-4-3-2-2-B-B-2 x (ICMR 312 S1-1-5-3-B x HHVBC)-7-1-1-1-B-B-B]-21-B-1-2 | 129 |
| 130 | {(MC 94 S1-34-1-B x HHVBC)-16-2-1-1-1-1-B-B-3 x (SRC II C3 S1-19-3-2 x HHVBC)-5-3-1-1-B-B-B}-29-2-1-3-3-5-3-1 | 130 |
| 131 | {(MC 94 S1-34-1-B x HHVBC)-12-1-2-2-2-B-1-B-B x [ICMB 95111 x (D2BLN/95-107 x EEBC C1-1)-6-B]-24-4-1-B-B-B-B}-4-2-2-4 | 131 |
| 132 | [(MC 94 S1-34-1-B x HHVBC)-16-1-3-1-2-2-B-B-2-B-B x ICMB 98222]-1-3-1-1 | 132 |
| 133 | (HTBLN/95-98 x ICMB 89111)-3-B-B-3-B-B-B-B-B | 133 |
| 134 | (SRC II C3 S1-19-3-2 x HHVBC)-3-5-1-1-2-B | 134 |
| 135 | {(MC 94 S1-81-1-B x HHVBC)-4-4-1 x (MC 94 S1-81-1-B x HHVBC)-4-2-4-10-3-1-B-B-B-2 | 135 |
| 136 | (B x B) F2 S1-109-2-3-3-1-1-4 | 136 |
| 137 | (B x B) F2 S1-109-2-3-3-1-4-2 | 137 |
| 138 | (ICMB 01888 x ICMB 01222)-4-2-B-2-B-B-1 | 138 |
| 139 | (ICMB 93333 x ICMB 01222)-11-2-1-B-B | 139 |
| 140 | (ICMB 93333 x ICMB 01222)-11-2-2-2-B-2-6 | 140 |
| 141 | (ICMB 93333 x ICMB 01222)-20-2-B-5-B-1-B | 141 |
| 142 | (ICMB 93333 x ICMB 01222)-20-2-B-5-B-4-B | 142 |
| 143 | (ICMB 94333 x ICMB 01222)-47-1-B-B-B | 143 |
| 144 | [{(ICMB 96555 x IP 10437)-9-B-B-B-B-B-B x IP 14758-2-2}-19-1-B x (ICMB 96555 x IP 10437)-3-4-1-2-2-1-B-2-B-3]-1-1-1-1-1 | 144 |
| 145 | [ICMB 97444 x (SPF3/S91-544 x (SPF3/91-5)-5-1-2-1)-5-1-B-B-B-B x ICMB 00444-4-3-1-3 | 145 |
| 146 | {NC D2 S1-1-2-1-2-3-1-B x [ICMB 95111 x (ICMB 94555 x D2BLN/95-75)-6-2-B-B]-47-2-1-B-B}-1-1-3-3 | 146 |
| 147 | IC-CZBC-C0-60-3-1-2-3 R5-3-B-B | 147 |
| 148 | ([ICMB 95111 x (ICMB 96555 x IP 10437)-3]-7-2-1-B-2-15-1] x B-bulk (3981-3989/S06 G1)}-3-2-4-B x HHVDBC HS-155-1-1-1-2-1-1-B)-3-1-5-3 | 148 |
| 149 | ([ICMB 95111 x (ICMB 96555 x IP 10437)-3]-7-2-1-B-2-15-1] x B-bulk (3981-3989/S06 G1)}-3-2-4-B x HHVDBC HS-155-1-1-1-2-1-1-B)-3-3-1-3 | 149 |
| 150 | (ICMB 04888 x HHVDBC HS-10-1-2-1-1-1-2-B)-12-3-6-4-4 | 150 |
| 151 | (ICMB 04888 x HHVDBC HS-10-1-2-1-1-1-2-B)-2-1-3-1 | 151 |
| 152 | (ICMB 04888 x HHVDBC HS-10-1-2-1-1-1-2-B)-2-1-4-3 | 152 |
| 153 | {[78-7088/3/SER3 AD//B282/(3/4)EB x PBLN/S95-359]-7-4-B-B-2-B-B x HHVDBC HS-10-1-2-1-1-4-1-2}-28-2-4-6 | 153 |
| 154 | (ICMB 03111 x [(MC 94 S1-34-1-B x HHVBC)-10-4-3-2-2-B-B-2 x (ICMR 312 S1-1-5-3-B x HHVBC)-7-1-1-1-B-B-B]-21-B-1-4-1)-18-2-1-2 | 154 |
| 155 | (ICMB 03111 x [(MC 94 S1-34-1-B x HHVBC)-10-4-3-2-2-B-B-2 x (ICMR 312 S1-1-5-3-B x HHVBC)-7-1-1-1-B-B-B]-21-B-1-4-1)-6-1-3-1 | 155 |
| 156 | (ICMB 03111 x {(MC 94 S1-34-1-B x HHVBC)-16-2-1-1-1-1-B-B-3 x (SRC II C3 S1-19-3-2 x HHVBC)-5-3-1-1-B-B-B}-29-2-1-3-1)-8-3-1-2 | 156 |
| 157 | (ICMB 03111 x {(MC 94 S1-34-1-B x HHVBC)-16-2-1-1-1-1-B-B-5 x (MC 94 S1-34-1-B x HHVBC)-10-4-1-2-1-B-B-1-30-2-4-2-1)-4-2-3-3 | 157 |
| 158 | (ICMB 03111 x {(MC 94 S1-34-1-B x HHVBC)-16-2-1-1-1-1-B-B-5 x (MC 94 S1-34-1-B x HHVBC)-10-4-1-2-1-B-B-1-30-2-4-2-1)-7-5-4-1-1 | 158 |
| 159 | (ICMB 03111 x {(MC 94 S1-34-1-B x HHVBC)-16-2-1-1-1-1-B-B-5 x (MC 94 S1-34-1-B x HHVBC)-10-4-1-2-1-B-B-1-30-2-4-3-1)-13-2-3-3 | 159 |
| 160 | (ICMB 03111 x {(MC 94 S1-34-1-B x HHVBC)-16-2-1-1-1-1-B-B-5 x (MC 94 S1-34-1-B x HHVBC)-10-4-1-2-1-B-B-1-30-2-4-3-1)-13-2-5-1 | 160 |
| 161 | MC 94 C2-S1-3-1-3-3-2-2-B | 161 |
| 162 | SDMV 90031-S1-3-3-2-1-3-2-2-1-B | 162 |
| 163 | SDMV 90031-S1-93-3-1-1-3-2-2-1-1-B | 163 |
| 164 | [((MC 94 S1-34-1-B x HHVBC)-16-2-1) × (IP 19626-4-2-3)]-B-37-1-1-1-2-B | 164 |
| 165 | AIMP 92901 S1-296-2-1-1-3-B-1-B-B | 165 |
| 166 | MRC HS-91-2-3-3-B-B-B-B-B | 166 |
| 167 | MC 94 C2-S1-47-1-1-3-B-1-B-B | 167 |
| 168 | MRC HS-219-2-1-2-B-B-B-B | 168 |
| 169 | MRC S1-4-1-3-B-B-B-B | 169 |
| 170 | MRC HS-130-6-1-1-B-B-B-B-B-B | 170 |
| 171 | JBV 3 S1-95-3-1-2-B | 171 |
| 172 | ICMV 91059 S1-20-1-2-1-4-1-B-B | 172 |
| 173 | ICMS 8511 S1-17-2-1-1-4-1-B-3-2-2-B | 173 |
| 174 | ICMS 7704-S1-126-5-2-1-3-2-2-2-B-3 | 174 |
| 175 | Jakhrana × ESRC II S2-81-3-2-2-2 | 175 |
| 176 | (RCB-2-S1-138-1-3 × MRC)-B-2-1-2-B | 176 |
| 177 | (ICMV-IS 94206-7 × (SRC II C3 S1-1-1-2 x HHVBC)-1-3-3))-B-10-1-2-2 | 177 |
| 178 | (ICMS 7704-S1-127-5-1 × RCB-2 Tall )-B-19-3-4-5-3 | 178 |
| 179 | ICMR 312 S1-8-1-1-1-1-B-B-B-1-B | 179 |
| 180 | RCB-2 S1-19-2-2-1-2-3-2-1-B-B-B | 180 |
| 181 | [(((ICMV-IS 94206-15)×B-Lines)-B-6) × (MRC S1-156-2-1-B)]-B-13-1-3-3-2-B | 181 |
| 182 | ICMV 91059 S1-4-2-3-2-1-1-4-B-1-5-B-B | 182 |
| 183 | HHVBC tall (C1) S1-33-3-1-1-1-2-B-B-3-2 | 183 |
| 184 | [((MC 94 S1-34-1-B x HHVBC)-16-2-1) × (IP 19626-4-2-3)]-B-18-2-2-4-1-B | 184 |
| 185 | ICMS 7704-S1-52-3-1-2-1-2-1-6-B-B | 185 |
| 186 | JBV 3 S1-18-2-2-1-3-2 | 186 |
| 187 | JBV 3 S1-6-1-2-1-2-3-B | 187 |
| 188 | ICMS 7704-S1-127-5-1-5-1-1-3-3-2-B-B | 188 |
| 189 | MRC HS-225-3-5-2-B-B-B-B-B | 189 |
| 190 | [(((ICMV-IS 94206-15)×B-Lines)-B-6) × (MRC S1-156-2-1-B)]-B-38-3-1-B-7-B | 190 |
| 191 | ICMS 7704-S1-80-2-1-1-2-2-1-B-B-B-B | 191 |
| 192 | [(((IP 12322-1-2)×B-Lines)-B-14) × (MRC S1-156-2-1-B)]-B-1-3-3-B-B | 192 |
| 193 | JBV 3 S1-35-2-1-2-B | 193 |
| 194 | [((MC 94 S1-34-1-B x HHVBC)-16-2-1) × (IP 19626-4-2-3)]-B-34-1-3-3-1-1-B-2 | 194 |
| 195 | JBV 3 S1-257-1-4-1-B | 195 |
| 196 | AIMP 92901 S1-480-1-1-2-3-1 | 196 |
| 197 | ((ICMV IS 94206 S1-15-2)×{(SRC II C3 S1-19-3-2 x HHVBC)-5-3-1})-B-13-4-2-1-1-1-1-3-2 | 197 |
| 198 | JBV 3 S1-2-3-2-2-B-2 | 198 |
| 199 | JBV 3 S1-6-1-2-1-2-2-B-B | 199 |
| 200 | ICMV 91059 S1-4-2-3-2-1-1-4-B-1-3-B-3 | 200 |
| 201 | ICMR 312 S1-17-3-2-1-2-2-B-1-B | 201 |
| 202 | MRC S1-97-3-4-B-B-1-B-B-B | 202 |
| 203 | AIMP 92901 S1-296-2-1-1-3-B-1-3-B-2 | 203 |
| 204 | ICMR 312 S1-17-3-2-1-2-3-B-B-B-B-B-B | 204 |
| 205 | JBV 3 S1-18-2-2-2-3-2-3-B | 205 |
| 206 | SDMV 90031-S1-3-3-2-1-3-2-2-3-1 | 206 |
| 207 | ICMV 91059 S1-4-2-3-2-1-1-4-B-1-3-B-1 | 207 |
| 208 | [(((IP 12322-1-2)×B-Lines)-B-8) × (MRC HS-170-3-5-2-B)]-B-5-3-2-3-B-B | 208 |
| 209 | SDMV 90031-S1-86-4-2-1-1-B-2-3-B-B-B | 209 |
| 210 | (EERC-HS-29)-B-12-4-1-1 | 210 |
| 211 | JBV 3 S1-33-2-1-3-3-B-3-B-1-B | 211 |
| 212 | (E 298 x LCSN 282-4-1-2)-12-2-1-2-B-B-B-1 | 212 |
| 213 | (ICMS 7704-S1-127-5-1 × RCB-2 Tall )-B-19-3-2-1-1-1-B | 213 |
| 214 | (MC 94 C2-S1-3-2-2-2-1-3-B-B x AIMP 92901 S1-488-2-1-1-4-B-B)-B-28-1-1 | 214 |
| 215 | [(IPC 1617×SDMV 90031-S1-84-1-1-1-1)×GB 8735-S1-25-4-4-1-1-3-1-1]-1-1-3-2-1-B-B | 215 |
| 216 | [(IPC 337×SDMV 90031-S1-84-1-1-1-1)×ICMS 8511 S1-14-3-1-1-2-B-1]-4-5-2-2-4-B-B | 216 |
| 217 | LaGrap C2-S1-14-4-3-4-1-B | 217 |
| 218 | (AIMP 92901 S1-480-1-1-1-2-B-2 x ICMR 312 S1-3-2-3-2-1-1-B-B)-B-11-1-1-B | 218 |
| 219 | (EERC-HS-32)-B-8-1-1-B | 219 |
| 220 | (EERC-HS-6)-B-4-2-3-3-B | 220 |
| 221 | (ICMV-IS 94206-17 × (SRC II C3 S1-1-1-2 x HHVBC)-1-3-3))-B-10-1-1-2-2-1-1-B | 221 |
| 222 | (IP 12370-1-3 × B-Lines)-B-9-1-2-1-2-2-4 | 222 |
| 223 | (IPC 107 ×ICMV 91059 S1-14-2-1-1-2)-13-2-2-2-1 | 223 |
| 224 | (IPC 1268 ×ICMV 91059 S1-58-2-2-2-1)-10-1-2-2-B | 224 |
| 225 | (IPC 1617 ×SDMV 90031-S1-84-1-1-1-1)-26-2-3-1 | 225 |
| 226 | (JBV 3 S1-197-1-1-1 x IPC 655(B))-B-14-3 | 226 |
| 227 | (MC 94 C2-S1-3-1-3-1-4-B-B x SDMV 93032-S1-5-2-1-1-6-B-B-B-2-B)-B-11-3 | 227 |
| 228 | (MC 94 C2-S1-3-2-2-2-1-3-B-B x ICMR 312 S1-3-2-3-2-1-1-B-B)-B-17-4-3-2 | 228 |
| 229 | (RCB-2-S1-43-3-4 × MRC)-B-2-1-1-B-1-B | 229 |
| 230 | (SDMV 90031 S1-3-3-2-1-3-1-1 x ICMS 8511 S1-14-3-1-1-2-B-1)-B-35-3-2-1-B-B | 230 |
| 231 | (SRC II C3 S1-19-3-2 x HHVBC)-1-5-1 x ICMB 04888)-B-3-2-3-1 | 231 |
| 232 | [(AIMP 92901 S1-488-2-1-1-2-B-1-B x R-lines bulk 20216-20249/K09)]-13-3 | 232 |
| 233 | [(IPC 1617×SDMV 90031-S1-84-1-1-1-1)×(ICMV-IS 94206-7 × (SRC II C3 S1-1-1-2 x HHVBC)-1-3-3))-B-3-1-1]-1-1-4-3-3 | 233 |
| 234 | [(IPC 337×SDMV 90031-S1-84-1-1-1-1)×ICMS 8511 S1-14-3-1-1-2-B-1]-3-2-4-2-3 | 234 |
| 235 | [(IPC 337×SDMV 90031-S1-84-1-1-1-1)×RCB-2-S1-144-2-2-2-1-1-1]-1-1-3-1 | 235 |
| 236 | [(Jakhrana × ESRC II S2-11-B-1-2-1-1-B) x (EERC-HS-23-B-3-1-2-4)]-7 | 236 |
| 237 | [(MRC S1-155-4-3-B-B-B-B-1-B-B-1)xHiTiP S1-7-1-1-3-1)]-2 | 237 |
| 238 | [ICMR 312 S1-3-2-1-2-4 x SDMV 93032-S1-5-2-1-1-6-B-B-B-2]-B-2 | 238 |
| 239 | [JBV 3 S1-300-1-1-2-2 x JBV 3 S1-18-1-3-3-2]-B-7-4x[((MC 94 S1-34-1-B x HHVBC)-16-2-1) × (IP 19626-4-2-3)]-B-4-1-3-3-1-2-1-1 | 239 |
| 240 | [MC 94 C2-S1-3-1-1-2-1-1-B-2 x IPC 1518]-B-5-1 | 240 |
| 241 | [MC 94 C2-S1-3-1-3-1-4-B-B x LaGrap C2-S1-97]-B-2-3 | 241 |
| 242 | {((MC 94 S1-34-1-B × HHVBC)-16-2-1) × (IP 19626-4-2-3)]-B-28-3-1-2-2}×{MRC HS 225-3-5-2-B-B-B-B}-B-4-2-2-1-B-B | 242 |
| 243 | {(SRC II C3 S1-19-3-2 × HHVBC)-1-5-1}×{[((96111B × 4017-6-1-1)-1-4-4-3) × (IP 19626-4-1-2-1)]-B-6}-B-5-1-1-3 | 243 |
| 244 | {[(((IP 12322-1-2)×B-Lines)-B-14) × (MRC S1-156-2-1-B)]-B-1-3-3-B}×{GB 8735-S1-15-3-1-1-3-4-2-2-2-1}-B-11-2-1-2-2 | 244 |
| 245 | {[ARD-288-1-10-1-2 (RM)-3 × B-lines bulk]-14-B-1-1}×{((ICMV IS 94206 S1-15-2)×{(SRC II C3 S1-19-3-2 × HHVBC)-5-3-1})-B-1-2-1-1}-B-8-1-2-2 | 245 |
| 246 | AIMP 92901 S1-15-1-2-3-B-1-B-14-3-1-B | 246 |
| 247 | AIMP 92901 S1-218-1-2-1-B-4-B | 247 |
| 248 | GB 8735-S1-15-3-1-1-3-3-1-1-2-1-3 | 248 |
| 249 | GB 8735-S1-25-4-4-1-1-3-3-3-4-B-1-B-B | 249 |
| 250 | ICMS 8506 S1-4-2-2-2-3-3-1-2-3-1-1-B-B | 250 |
| 251 | ICMV 91059 S1-58-4-4-2-4-5-B-B-B-1-B-B-B | 251 |
| 252 | IP No. 17645-1-1-1-1 | 252 |
| 253 | IP No. 8047-2-3-1-1 | 253 |
| 254 | IP No. 9348-1-1-2-2 | 254 |
| 255 | Jakhrana × ESRC II S2-11-B-1-2-1-1-B-B | 255 |
| 256 | Jakhrana × SRC II S2-215-3-2-1-B-3 | 256 |
| 257 | JBV 3 S1-131-3-1-2-3-1-B-1-B-B | 257 |
| 258 | JBV 3 S1-300-1-1-2-1-4-1 | 258 |
| 259 | JBV 3 S1-305-2-B-1-B-B-B | 259 |
| 260 | JBV 3 S1-334-1-2-3-2-B | 260 |
| 261 | JBV 3 S1-44-3-B-1-3 | 261 |
| 262 | LaGrap C2-S1-81-1-2-1-4-2 | 262 |
| 263 | MC 94 C2-S1-3-1-1-1-2-4-B-B-B-B | 263 |
| 264 | MDMRRC S1-1-136-1-2-1-B | 264 |
| 265 | MDMRRC S1-1-59-3-3-1-B | 265 |
| 266 | MDMRRC S1-329-1 | 266 |
| 267 | MDRRC-HS-28-2-3 | 267 |
| 268 | MRC HS-170-3-5-2-B-B-2-1-B-B | 268 |
| 269 | MRC HS-176-2-2-1-B-B-2-1-BxMRC S1-54-2-3-B-B-1-B-B-1 | 269 |
| 270 | MRC S1-155-4-3-B-B-B-B-1-B-B xR-lines bulk (20275-20291) | 270 |
| 271 | MRC S1-156-1-1-1-B-3-B-B-B-8-1-1-B | 271 |
| 272 | MRC S1-340-1-3-3-2-B-B-1 | 272 |
| 273 | MRC S1-416-2-1-2-3-B-B-B-1-B-B | 273 |
| 274 | MRC S1-452-5-2-B-B-B-B-B-B-B-B | 274 |
| 275 | MRC S1-9-2-2-B-B-2-B-B | 275 |
| 276 | RCB-2 S1-33-1-3-3-2-3-B-B-B-B-B-B-B | 276 |
| 277 | SDMV 90031-S1-60-1-1-1-5-2-B-B | 277 |
| 278 | SDMV 95045 S1-7-2-4-2-3-2-1-B-B-B-B-8-1-1 | 278 |
| 279 | (NEP 18-5025 x Serere 2A-2-2)-7-1-3-8 | 279 |
| 280 | (B 282 x 3/4Souna-100)-11-9-4 (Duplicate 000374) | 280 |
| 281 | 8102-2-2-3-3 (Duplicate 000756) | 281 |
| 282 | R Line | 282 |
| 283 | [(SOSAT-C 88-S1-60-B-B-1-2-1-3-1-2-1-3-2-1 x IP No 17843-1-3)]-3 | 283 |
| 284 | [(SOSAT-C 88-S1-60-B-B-1-2-1-3-1-2-1-3-2-1 x IP No 17843-1-3)]-3 | 284 |
| 285 | RCB-2 S1-19-2-5-3-2-3-1-1-B-1-1-B | 285 |
| 286 | (J 834 x 700516)-1-4-4-2-4-B-1 | 286 |
| 287 | 31923/S09/HP | 287 |
| 288 | Jakhrana × ESRC II S2-147-B-2-2-3-1-B | 288 |
| 289 | MRC S1-9-1-1-B-B-B-B-B-B (ISK sel)-1 | 289 |
| 290 | MRC S1-155-4-3-B-B-B-B-1-B-B-1 | 290 |
| 291 | MRC S1-340-1-3-2-B-4-B-B-B-B-B-1-B | 291 |
| 292 | (EERC-HS-23)-31-2-2-B-2-B | 292 |
| 293 | (EERC-HS-8)-3-2-1-B-B-3 | 293 |
| 294 | (MC 94 C2-S1-3-2-2-2-1-3-B-B x ICMR 312 S1-3-2-3-2-1-1-B-B)-B-41-1-1-1-B-B-B | 294 |
| 295 | {[((MC 94 S1-34-1-B x HHVBC)-16-2-1) × (IP 19626-4-2-3)]-B-28-3-2-2-2}×{GB 8735-S1-15-3-1-1-3-4-2-2-2-1}-B-30-2-2-1-B-B-B-1 | 295 |
| 296 | [ICMR 312 S1-3-2-1-2-4 x Lagrap C2 S1-11-P1]-B-1-3-1-2-B | 296 |
| 297 | [ICMR 312 S1-3-2-1-2-4 x Lagrap C2 S1-11-P1]-B-14-2-1-1-B | 297 |
| 298 | [ICMR 312 S1-3-2-1-2-4 x Lagrap C2 S1-11-P1]-B-15-1-2-2-B | 298 |
| 299 | [ICMR 312 S1-3-2-1-2-4 x Lagrap C2 S1-11-P1]-B-4-2-2-2-B | 299 |
| 300 | [ICMR 312 S1-3-2-1-2-4 x LaGrap C2-S1-64]-B-9-2-1-1-B | 300 |
| 301 | [ICMR 312 S1-3-2-1-2-4 x SDMV 93032-S1-5-2-1-1-6-B-B-B-2]-B-26-1-1-B | 301 |
| 302 | [MC 94 C2-S1-3-1-1-2-1-1-B-2 x IPC 1518]-B-5-3-1-1-B | 302 |
| 303 | [MC 94 C2-S1-3-1-3-1-4-B-B x LaGrap C2-S1-97]-B-11-1-1-2-B | 303 |
| 304 | [MC 94 C2-S1-3-1-3-1-4-B-B x LaGrap C2-S1-97]-B-3-1-2-1-B | 304 |
| 305 | {[((MC 94 S1-34-1-B x HHVBC)-16-2-1) × (IP 19626-4-2-3)]-B-18-2-3-2-2-BxAIMP 92901 S1-296-2-1-1-2-2}-B-25-3-3-3-B | 305 |
| 306 | {[((MC 94 S1-34-1-B x HHVBC)-16-2-1) × (IP 19626-4-2-3)]-B-18-2-3-2-2-BxAIMP 92901 S1-296-2-1-1-3-B-1-6-B-B}-B-14-4-3-1-B | 306 |
| 307 | {[((MC 94 S1-34-1-B x HHVBC)-16-2-1) × (IP 19626-4-2-3)]-B-28-2-2-3-1-2xICMR 312 S1-3-2-3-2-1-1-B-B-B-B}-B-17-2-1-3-B | 307 |
| 308 | [((SRC II C3 S1-19-3-2 × HHVBC)-3-5-1) × (IP 19626-4-1-1-1)]-B-2-2-1-1-1-4-6-1-5-3-B | 308 |
| 309 | [((SRC II C3 S1-19-3-2 × HHVBC)-3-5-1) × (IP 19626-4-1-1-1)]-B-2-2-1-1-2-2-2-2-4-3-B | 309 |
| 310 | [((SRC II C3 S1-19-3-2 × HHVBC)-3-5-1) × (IP 19626-4-1-1-1)]-B-2-2-1-1-4-2-1-3-2-1-B | 310 |
| 311 | [((SRC II C3 S1-19-3-2 × HHVBC)-3-5-1) × (IP 19626-4-1-1-1)]-B-2-2-1-2-2-2-2-1-2-1-B | 311 |
| 312 | [((SRC II C3 S1-19-3-2 × HHVBC)-3-5-1) × (IP 19626-4-1-1-1)]-B-2-2-1-2-4-2-B-3-1-1-B | 312 |
| 313 | {(SRC II C3 S1-19-3-2 × HHVBC)-1-5-1}×{[((96111B × 4017-6-1-1)-1-4-4-3) × (IP 19626-4-1-2-1)]-B-6}-B-5-2-3-1-B-1-B | 313 |
| 314 | {(SRC II C3 S1-19-3-2 × HHVBC)-1-5-1}×{[((96111B × 4017-6-1-1)-1-4-4-3) × (IP 19626-4-1-2-1)]-B-6}-B-5-2-3-1-B-3-B | 314 |
| 315 | MDMRRC S1-1-103-3-2-1-B | 315 |
| 316 | MDMRRC S1-1-120-1-2-1-B | R316 |
| 317 | MDMRRC S1-1-217-2-1-1-B | 317 |
| 318 | MDMRRC S1-1-276-1-2-1-1 | 318 |
| 319 | MDMRRC S1-1-278-2-5-3-B | 319 |
| 320 | MDMRRC S1-1-303-2-2-3-1 | 320 |
| 321 | MDMRRC S1-1-41-1-4-2-B | 321 |
| 322 | MDMRRC S1-1-42-2-2-2-2 | 322 |
| 323 | (MC 94 C2-S1-3-2-2-2-1-3-B-B x AIMP 92901 S1-488-2-1-1-4-B-B)-B-30-1-2-2 | 323 |
| 324 | (MC 94 C2-S1-3-2-2-2-1-3-B-B x AIMP 92901 S1-488-2-1-1-4-B-B)-B-30-3-4-2 | 324 |
| 325 | (MC 94 C2-S1-3-2-2-2-1-3-B-B x ICMR 312 S1-3-2-3-2-1-1-B-B)-B-32-3-3-5 | 325 |
| 326 | (MC 94 C2-S1-3-2-2-2-1-3-B-B x ICMR 312 S1-3-2-3-2-1-1-B-B)-B-34-4-1-2 | 326 |
| 327 | ICTP 8202 S1-56-1 | 327 |
| 328 | ICTP 8202 S1-25-1 | 328 |
| 329 | ICTP 8202-S1-28-1 | 329 |
| 330 | [(IPC 1268×ICMV 91059 S1-58-2-2-2-1)×AIMP 92901 S1-296-2-1-1-1-B-B]-2-2-3-1-2 | 330 |
| 331 | [(IPC 1268×ICMV 91059 S1-58-2-2-2-1)×AIMP 92901 S1-296-2-1-1-1-B-B]-2-2-3-2-3 | 331 |
| 332 | [(IPC 1268×ICMV 91059 S1-58-2-2-2-1)×AIMP 92901 S1-296-2-1-1-1-B-B]-4-2-1-3-3 | 332 |
| 333 | ICMS 7704-S1-126-5-2-2-5-1-3-B-B-1-17 | 333 |
| 334 | (ICMV-IS 94206-7 × (SRC II C3 S1-1-1-2 x HHVBC)-1-3-3))-B-10-1-1-5-4-1-2-6 | 334 |
| 335 | (IPC 107 ×SDMV 90031-S1-84-1-1-1-1)-1-2-3-1-1 | 335 |
| 336 | (IPC 1617 ×SDMV 90031-S1-84-1-1-1-1)-25-2-4-2-B-B | 336 |
| 337 | HiTiP S1-7-1-2-2-1-B | 337 |
| 338 | MRC HS-86-1-1-5-B-B-B-B-BxMRC S1-214-2-5-B-B-B-B-B-1-B | 338 |
| 339 | {MRC S1-9-2-2-B-B-4-B-B-B-B x (ICMS 7704-S1-127-5-1 × RCB-2 Tall )-B-19-3-3-5-1}-1-3-B | 339 |
| 340 | (MC 94 C2-S1-3-1-3-1-4-B-B x SDMV 93032-S1-5-2-1-1-6-B-B-B-2-B)-B-17-1-1 | 340 |
| 341 | (MC 94 C2-S1-3-2-2-2-1-3-B-B x AIMP 92901 S1-488-2-1-1-4-B-B)-B-8-2-2 | 341 |
| 342 | (IPC 107 ×SDMV 90031-S1-84-1-1-1-1)-1-1-1-3-BxICMR 312 S1-3-2-3-2-1-1-B-1 | 342 |
| 343 | Tift23 D_2_B_1_P1-P5 | Check |
| 344 | Tift23 D_2_B_1_P1-P5 | Check |
| 345 | Tift23 D_2_B_1_P1-P5 | Check |
| 346 | Tift23 D_2_B_1_P1-P5 | Check |
| 347 | Tift23 D_2_B_1_P1-P5 | Check |

Note: Tift23 D_2_B_1_P1-P5 was repeated five times.
