# Supplementary material for: Towards Defining Heterotic Gene Pools in Pearl Millet [Pennisetum glaucum (L.) R. Br.]
Source: Front Plant Sci. 2018 Mar 2;8:1934. doi: 10.3389/fpls.2017.01934 (PMC5841052; doi:10.3389/fpls.2017.01934)
Supplement: Supplementary file 2 [file Table2.DOCX]

**Table S2: Details of SSR markers used for the study**

| **S.No.** | **Marker** | **Motif** | **Observed band size (bp)** | **Expected band size (bp)** | **Linkage group (LG)** |
| --- | --- | --- | --- | --- | --- |
| 1 | Xicmp3002 | (CGTG)5 | 225-240 | NA | 6 |
| 2 | Xicmp3050 | (TA)8 | 214-226 | 214 | 6 |
| 3 | Xicmp3086 | (CAT)5 | 141-150 | 153 | 6 |
| 4 | Xipes0004 | (GT)11 | 119-131 | 116/130-142 | 1 |
| 5 | Xipes0007 | (CTG)11 | 127-142 | 120/127-143 | 2 |
| 6 | Xipes0011 | (TGGAG)4 | 198-208 | 187/204-215 | 6 |
| 7 | Xipes0014 | (CTG)8 | 184-196 | 172/184-196 | NA |
| 8 | Xipes0015 | (AT)11 | 105-113 | 104/108-120 | 7 |
| 9 | Xipes0017 | (TA)10 | 139-173 | 132/137-156 | 1 |
| 10 | Xipes0026 | (CGTATC)4 | 128-134 | 115/127-140 | 7 |
| 11 | Xipes0027 | (GTT)8 | 229-304 | 214/230-303 | 6 or 2 |
| 12 | Xipes0035 | (TGG)8 | 144-165 | 147/151-169 | 6 |
| 13 | Xipes0045 | (CTG)9 | 202-211 | 194/203-212 | 1 |
| 14 | Xipes0066 | (GT)10 | 108-122 | 101/113-121 | 4 |
| 15 | Xipes0071 | (TATG)5 | 183-191 | 175/180-195 | 6 |
| 16 | Xipes0079 | (AGAAA)4 | 235-240 | 213/226-239 | 1 |
| 17 | Xipes0082 | (AGGAG)7 | 168-183 | 161/166-181 | 7 |
| 18 | Xipes0087 | (AGA)7 | 364-379 | 351/361-381 | 6 |
| 19 | Xipes0089 | (GCT)8 | 349-364 | 340/356-365 | 5 |
| 20 | Xipes0093 | (TGA)10 | 142-154 | 134/138-160 | 5 |
| 21 | Xipes0095 | (GAG)7 | 199-205 | 201/211-230 | 3 |
| 22 | Xipes0097 | (CCAT)5 | 214-226 | 201/212-226 | 7 |
| 23 | Xipes0098 | (TGA)12 | 115-160 | 110/133-156 | 1 |
| 24 | Xipes0101 | (GGAGCC)5 | 309-363 | 339/343-360 | 1 |
| 25 | Xipes0103 | (GGA)7 | 290-296 | 277/289-295 | 1 |
| 26 | Xipes0109 | (TTGCA)4 | 259-279 | 237/254-274 | 6 |
| 27 | Xipes0110 | (ATGT)5 | 258-282 | 246/264-275 | NA |
| 28 | Xipes0118 | (CTCCAG)4 | 166-172 | 152/134-176 | 2 |
| 29 | Xipes0126 | (AGC)8 | 116-134 | 106/113-130 | 1 |
| 30 | Xipes0127 | (TGCTT)5 | 289-324 | 203/284-305 | 1 |
| 31 | Xipes0129 | (CACAG)6 | 142-162 | 147/159-180 | 4 |
| 32 | Xipes0141 | (TTGCA)5 | 155-175 | 140/154-175 | 6 |
| 33 | Xipes0142 | (AACTC)4 | 164-174 | 155/161-175 | 3 |
| 34 | Xipes0144 | (CTGTG)5 | 123-148 | 123/124-146 | 6 |
| 35 | Xipes0145 | (CATCC)6 | 233-253 | 219/235-241 | 7 |
| 36 | Xipes0146 | (ATCTTC)6 | 146-164 | 149/156-171 | 1 |
| 37 | Xipes0147 | (CAG)7 | 172-175 | 153/167-176 | 6 |
| 38 | Xipes0151 | (TCGA)5 | 163-171 | 150/166-176 | 6 |
| 39 | Xipes0153 | (GCGAT)5 | 169-179 | 167/179-185 | 7 |
| 40 | Xipes0154 | (GT)11 | 321-341 | 309/317-339 | 7 |
| 41 | Xipes0156 | (TTC)9 | 233-254 | 237/252-269 | 6 |
| 42 | Xipes0160 | (AC)10 | 142-150 | 134/140-153 | 2 |
| 43 | Xipes0162 | (AAC)8 | 288-294 | 263/307-315 | 2 |
| 44 | Xipes0166 | (GAC)14 | 236-257 | 242/237-260 | 3 |
| 45 | Xipes0174 | (CCGT)5 | 200-216 | 248/259-270 | 4 |
| 46 | Xipes0176 | (TGC)7 | 363-375 | 367/381-390 | 6 |
| 47 | Xipes0179 | (TGGAC)5 | 269-289 | 257/270-281 | 7 |
| 48 | Xipes0180 | (TGTAT)4 | 331-351 | 320/338-349 | 3 |
| 49 | Xipes0181 | (GCT)8 | 217-229 | 202/213-228 | 2 |
| 50 | Xipes0186 | (TTG)10 | 261-273 | 248/262-272 | 4 |
| 51 | Xipes0189 | (GATG)5 | 335-351 | 316/334-354 | 6 |
| 52 | Xipes0192 | (ATGT)6 | 267-327 | 270/283-292 | 1 |
| 53 | Xipes0197 | (GTGTA)5 | 286-306 | 286/286-311 | 1 |
| 54 | Xipes0198 | (AATACC)8 | 276-324 | 196/278-296 | 7 |
| 55 | Xipes0200 | (GTAC)11 | 183-211 | 192/190-211 | 6 |
| 56 | Xipes0203 | (ATC)16 | 258-303 | 256/239-278 | 1 |
| 57 | Xipes0205 | (GCGGT)4 | 409-414 | 392/408-419 | 7 |
| 58 | Xipes0206 | (AGC)4 | 329-377 | 347/355-367 | 7 |
| 59 | Xipes0207 | (TG)8(GC) | 131-149 | 126/136-152 | 6 |
| 60 | Xipes0208 | (CTC)4TA(CTA)4 | 285-321 | 299/295-323 | 4 |
| 61 | Xipes0210 | (AT)6 | 179-187 | 176/195-205 | 2 |
| 62 | Xipes0213 | (GAT)5 | 154-163 | 154/169-177 | 3 |
| 63 | Xipes0214 | (AGA)5 | 240-246 | 238/252-260 | 5 |
| 64 | Xipes0218 | (AT)6 | 250-258 | 244/260-270 | 2 |
| 65 | Xipes0219 | TTT(CT)6TT | 152-160 | 139/153-164 | 4 |
| 66 | Xipes0220 | (AG)5(TG)7 | 175-193 | 165/178-191 | 5 or 3 |
| 67 | Xipes0221 | (TA)6 | 286-294 | 266/281-290 | 2 |
| 68 | Xipes0223 | (AT)8 | 110-118 | 102/117-129 | 5 |
| 69 | Xipes0225 | (GATC)3 | 303-311 | 290/303-309 | 4 |
| 70 | Xipes0226 | (AGC)8 | 394-412 | 386/392-409 | 1 |
| 71 | Xipes0227 | (GAT)5 | 258-267 | 244/250-266 | 6 |
| 72 | Xipes0233 | (ATT)15 | 215-293 | 210/224-267 | 3 |
| 73 | Xpsmp2040 | (CA)nd | 160-178 | 163 | 7 |
| 74 | Xpsmp2070 | (CA)25(TA)6 | 188-262 | 226 | 3 |
| 75 | Xpsmp2077 | (CA)15(TA)8 | 137-175 | 180 | 2 |
| 76 | Xpsmp2201 | (GT)6 | 331-367 | 364 | 2 |
| 77 | Xpsmp2203 | (GT)18imperfect | 352-376 | 357 | 7 |
| 78 | Xpsmp2204 | (GT)7 | 264-268 | 266 | NA |
| 79 | Xpsmp2206 | (GT)11 | 204-218 | 203 | 2 |
| 80 | Xpsmp2208 | (GT)10 | 249-317 | 253 | 5 |
| 81 | Xpsmp2214 | (GT)9 | 244-248 | 246 | 3 |
| 82 | Xpsmp2215 | (GT)6 | 244-252 | 238 | NA |
| 83 | Xpsmp2227 | (GT)7 | 192-212 | 197 | 3 |
| 84 | Xpsmp2235 | (TG)9 | 182-184 | 192 | NA |
| 85 | Xpsmp2236 | (TG)4(GT)4 | 262-266 | 265 | 7 |
| 86 | Xpsmp2237 | (GT)8 | 212-258 | 233 | 2 |
| 87 | Xpsmp2240 | (TG)5 | 146-150 | 147 | NA |
| 88 | Xpsmp2253 | (TG)11 | 135-137 | 159 | NA |
