# Supplementary material for: Towards Defining Heterotic Gene Pools in Pearl Millet [Pennisetum glaucum (L.) R. Br.]
Source: Front Plant Sci. 2018 Mar 2;8:1934. doi: 10.3389/fpls.2017.01934 (PMC5841052; doi:10.3389/fpls.2017.01934)
Supplement: Supplementary file 3 [file Table3.DOCX]

**Table S3. Summary statistics of the 88 SSR markers used for genotyping 165 maintainer (B-) lines and 182 restorer (R-) lines of pearl millet**

| **S. No.** | **Marker** | **Allele Number** | **Availability** | **Gene Diversity** | **Heterozygosity** | **PIC** |
| --- | --- | --- | --- | --- | --- | --- |
| 1 | Xicmp3002 | 6 (6, 6) | 0.95 (0.95, 0.95) | 0.66 (0.65, 0.66) | 0.02 (0.01, 0.03) | 0.60 (0.59, 0.60) |
| 2 | Xicmp3050 | 6 (6, 4) | 0.73 (0.79, 0.68) | 0.47 (0.46, 0.47) | 0.02 (0.01, 0.04) | 0.44 (0.43, 0.43) |
| 3 | Xicmp3086 | 3 (3, 3) | 0.95 (0.95, 0.95) | 0.30 (0.31, 0.28) | 0.02 (0.00, 0.03) | 0.28 (0.29, 0.26) |
| 4 | Xipes0004 | 6 (5, 6) | 0.88 (0.93, 0.84) | 0.72 (0.69, 0.69) | 0.01 (0.00, 0.03) | 0.67 (0.64, 0.65) |
| 5 | Xipes0007 | 6 (6, 4) | 0.99 (0.98, 0.99) | 0.45 (0.43, 0.45) | 0.02 (0.02, 0.02) | 0.40 (0.40, 0.39) |
| 6 | Xipes0011 | 3 (3, 2) | 0.77 (0.82, 0.71) | 0.24 (0.27, 0.20) | 0.01 (0.01, 0.01) | 0.21 (0.24, 0.18) |
| 7 | Xipes0014 | 5 (5, 5) | 0.90 (0.95, 0.86) | 0.76 (0.73, 0.74) | 0.02 (0.02, 0.03) | 0.72 (0.69, 0.70) |
| 8 | Xipes0015 | 5 (5, 5) | 0.99 (0.97, 1.00) | 0.61 (0.57, 0.64) | 0.03 (0.02, 0.04) | 0.55 (0.50, 0.58) |
| 9 | Xipes0017 | 15 (10, 9) | 0.94 (0.96, 0.93) | 0.64 (0.60, 0.66) | 0.02 (0.01, 0.02) | 0.59 (0.52, 0.62) |
| 10 | Xipes0026 | 2 (2, 0) | 1.00 (1.00, 0.99) | 0.50 (0.45, 0.43) | 0.02 (0.02, 0.02) | 0.37 (0.35, 0.34) |
| 11 | Xipes0027 | 17 (12, 15) | 0.87 (0.85, 0.88) | 0.84 (0.73, 0.88) | 0.07 (0.06, 0.09) | 0.83 (0.70, 0.87) |
| 12 | Xipes0035 | 7 (6, 7) | 0.72 (0.84, 0.61) | 0.76 (0.68, 0.81) | 0.00 (0.01, 0.00) | 0.72 (0.63, 0.78) |
| 13 | Xipes0045 | 4 (3, 4) | 0.98 (0.98, 0.97) | 0.19 (0.20, 0.19) | 0.00 (0.00, 0.00) | 0.18 (0.18, 0.18) |
| 14 | Xipes0066 | 9 (5, 9) | 0.97 (0.99, 0.95) | 0.59 (0.31, 0.73) | 0.04 (0.01, 0.06) | 0.55 (0.30, 0.69) |
| 15 | Xipes0071 | 3 (3, 3) | 0.93 (0.93, 0.93) | 0.63 (0.66, 0.57) | 0.02 (0.01, 0.04) | 0.55 (0.58, 0.48) |
| 16 | Xipes0079 | 2 (2, 2) | 0.99 (0.99, 0.99) | 0.41 (0.41, 0.40) | 0.01 (0.00, 0.01) | 0.32 (0.33, 0.32) |
| 17 | Xipes0082 | 4 (4, 4) | 0.96 (0.96, 0.96) | 0.54 (0.57, 0.35) | 0.03 (0.02, 0.03) | 0.45 (0.50, 0.30) |
| 18 | Xipes0087 | 5 (5, 5) | 0.99 (0.99, 0.98) | 0.68 (0.56, 0.75) | 0.04 (0.02, 0.06) | 0.63 (0.48, 0.72) |
| 19 | Xipes0089 | 5 (5, 4) | 0.99 (0.99, 0.98) | 0.61 (0.46, 0.64) | 0.03 (0.02, 0.04) | 0.54 (0.42, 0.58) |
| 20 | Xipes0093 | 6 (6, 5) | 0.75 (0.76, 0.75) | 0.66 (0.55, 0.52) | 0.02 (0.02, 0.01) | 0.61 (0.51, 0.48) |
| 21 | Xipes0095 | 3 (2, 3) | 0.91 (0.91, 0.92) | 0.29 (0.30, 0.29) | 0.00 (0.00, 0.00) | 0.26 (0.26, 0.26) |
| 22 | Xipes0097 | 4 (4, 4) | 0.99 (1.00, 0.99) | 0.40 (0.34, 0.45) | 0.02 (0.01, 0.03) | 0.36 (0.31, 0.40) |
| 23 | Xipes0098 | 16 (11, 14) | 0.82 (0.88, 0.77) | 0.84 (0.81, 0.85) | 0.00 (0.00, 0.00) | 0.82 (0.79, 0.62) |
| 24 | Xipes0101 | 5 (5, 5) | 0.87 (0.90, 0.84) | 0.72 (0.68, 0.74) | 0.01 (0.01, 0.02) | 0.67 (0.62, 0.70) |
| 25 | Xipes0103 | 3 (3, 3) | 0.97 (0.99, 0.96) | 0.28 (0.30, 0.27) | 0.01 (0.00, 0.02) | 0.26 (0.27, 0.25) |
| 26 | Xipes0109 | 5 (5, 4) | 0.85 (0.85, 0.86) | 0.70 (0.64, 0.72) | 0.03 (0.01, 0.04) | 0.65 (0.59, 0.67) |
| 27 | Xipes0110 | 6 (4, 6) | 0.96 (0.93, 0.99) | 0.62 (0.67, 0.47) | 0.03 (0.03, 0.03) | 0.57 (0.60, 0.44) |
| 28 | Xipes0118 | 3 (3, 2) | 0.94 (0.95, 0.93) | 0.50 (0.46, 0.48) | 0.05 (0.04, 0.05) | 0.38 (0.36, 0.36) |
| 29 | Xipes0126 | 7 (6, 6) | 0.96 (0.95, 0.96) | 0.67 (0.62, 0.69) | 0.03 (0.01, 0.05) | 0.62 (0.58, 0.63) |
| 30 | Xipes0127 | 4 (4, 3) | 0.97 (0.97, 0.96) | 0.65 (0.67, 0.62) | 0.03 (0.03, 0.03) | 0.58 (0.59, 0.54) |
| 31 | Xipes0129 | 5 (3, 4) | 0.97 (0.99, 0.95) | 0.50 (0.47, 0.52) | 0.03 (0.02, 0.05) | 0.40 (0.39, 0.41) |
| 32 | Xipes0141 | 5 (5, 4) | 0.87 (0.86, 0.88) | 0.67 (0.60, 0.71) | 0.02 (0.01, 0.02) | 0.62 (0.55, 0.66) |
| 33 | Xipes0142 | 2 (2, 2) | 0.99 (0.99, 0.99) | 0.17 (0.07, 0.25) | 0.03 (0.01, 0.04) | 0.16 (0.07, 0.22) |
| 34 | Xipes0144 | 6 (4, 6) | 0.95 (0.93, 0.97) | 0.61 (0.52, 0.67) | 0.05 (0.01, 0.07) | 0.56 (0.46, 0.62) |
| 35 | Xipes0145 | 5 (4, 4) | 0.97 (0.97, 0.96) | 0.57 (0.38, 0.67) | 0.02 (0.01, 0.03) | 0.52 (0.34, 0.61) |
| 36 | Xipes0146 | 4 (3, 3) | 0.86 (0.88, 0.84) | 0.31 (0.20, 0.39) | 0.01 (0.02, 0.00) | 0.26 (0.18, 0.32) |
| 37 | Xipes0147 | 2 (1, 2) | 0.79 (0.85, 0.74) | 0.02 (0.00, 0.04) | 0.00 (0.00, 0.00) | 0.02 (0.00, 0.04) |
| 38 | Xipes0151 | 3 (3, 3) | 0.70 (0.76, 0.64) | 0.13 (0.05, 0.22) | 0.02 (0.00, 0.03) | 0.13 (0.05, 0.21) |
| 39 | Xipes0153 | 3 (3, 3) | 0.71 (0.65, 0.76) | 0.33 (0.23, 0.41) | 0.03 (0.05, 0.02) | 0.31 (0.22, 0.37) |
| 40 | Xipes0154 | 11 (10, 10) | 0.97 (0.96, 0.97) | 0.76 (0.61, 0.75) | 0.06 (0.06, 0.06) | 0.73 (0.59, 0.73) |
| 41 | Xipes0156 | 8 (8, 7) | 0.81 (0.85, 0.77) | 0.79 (0.78, 0.74) | 0.00 (0.00, 0.01) | 0.76 (0.76, 0.70) |
| 42 | Xipes0160 | 5 (5, 5) | 0.76 (0.72, 0.81) | 0.66 (0.52, 0.70) | 0.00 (0.00, 0.00) | 0.61 (0.47, 0.65) |
| 43 | Xipes0162 | 3 (3, 2) | 0.75 (0.76, 0.74) | 0.29 (0.45, 0.11) | 0.00 (0.00, 0.00) | 0.27 (0.40, 0.11) |
| 44 | Xipes0166 | 6 (6, 5) | 0.81 (0.63, 0.97) | 0.71 (0.61, 0.68) | 0.04 (0.04, 0.04) | 0.66 (0.53, 0.62) |
| 45 | Xipes0174 | 5 (4, 5) | 0.94 (0.93, 0.95) | 0.51 (0.53, 0.46) | 0.00 (0.00, 0.00) | 0.46 (0.47, 0.39) |
| 46 | Xipes0176 | 5 (5, 3) | 0.72 (0.72, 0.71) | 0.65 (0.63, 0.56) | 0.00 (0.00, 0.00) | 0.58 (0.55, 0.46) |
| 47 | Xipes0179 | 5 (4, 4) | 0.69 (0.56, 0.81) | 0.63 (0.63, 0.62) | 0.02 (0.02, 0.03) | 0.58 (0.57, 0.57) |
| 48 | Xipes0180 | 5 (3, 5) | 0.99 (1.00, 0.98) | 0.66 (0.45, 0.69) | 0.06 (0.02, 0.08) | 0.60 (0.41, 0.63) |
| 49 | Xipes0181 | 4 (3, 4) | 0.98 (0.96, 0.99) | 0.52 (0.52, 0.50) | 0.04 (0.02, 0.06) | 0.47 (0.43, 0.46) |
| 50 | Xipes0186 | 5 (3, 5) | 0.96 (0.92, 0.99) | 0.58 (0.41, 0.62) | 0.05 (0.03, 0.07) | 0.50 (0.35, 0.55) |
| 51 | Xipes0189 | 5 (5, 5) | 0.80 (0.87, 0.73) | 0.69 (0.63, 0.70) | 0.01 (0.01, 0.02) | 0.64 (0.56, 0.66) |
| 52 | Xipes0192 | 13 (10, 12) | 0.92 (0.95, 0.90) | 0.70 (0.52, 0.77) | 0.01 (0.01, 0.01) | 0.67 (0.50, 0.74) |
| 53 | Xipes0197 | 3 (3, 3) | 0.90 (0.90, 0.90) | 0.60 (0.59, 0.60) | 0.04 (0.04, 0.04) | 0.53 (0.51, 0.53) |
| 54 | Xipes0198 | 9 (9, 8) | 0.94 (0.92, 0.97) | 0.76 (0.65, 0.80) | 0.07 (0.03, 0.10) | 0.73 (0.62, 0.77) |
| 55 | Xipes0200 | 9 (9, 7) | 0.95 (0.97, 0.93) | 0.78 (0.76, 0.78) | 0.02 (0.02, 0.03) | 0.75 (0.72, 0.75) |
| 56 | Xipes0203 | 15 (12, 15) | 0.92 (0.95, 0.89) | 0.83 (0.77, 0.85) | 0.05 (0.02, 0.09) | 0.81 (0.74, 0.84) |
| 57 | Xipes0205 | 2 (2, 2) | 0.94 (0.93, 0.95) | 0.23 (0.25, 0.22) | 0.00 (0.00, 0.01) | 0.20 (0.22, 0.19) |
| 58 | Xipes0206 | 12 (6, 12) | 0.95 (1.00, 0.91) | 0.76 (0.69, 0.82) | 0.07 (0.03, 0.11) | 0.73 (0.63, 0.80) |
| 59 | Xipes0207 | 6 (5, 6) | 0.85 (0.86, 0.84) | 0.57 (0.37, 0.68) | 0.04 (0.06, 0.03) | 0.52 (0.34, 0.63) |
| 60 | Xipes0208 | 8 (8, 7) | 0.97 (0.97, 0.96) | 0.72 (0.65, 0.74) | 0.02 (0.00, 0.02) | 0.68 (0.60, 0.70) |
| 61 | Xipes0210 | 5 (5, 4) | 0.93 (0.92, 0.94) | 0.55 (0.48, 0.59) | 0.00 (0.00, 0.00) | 0.48 (0.42, 0.51) |
| 62 | Xipes0213 | 4 (4, 3) | 0.71 (0.77, 0.65) | 0.50 (0.43, 0.52) | 0.01 (0.01, 0.01) | 0.41 (0.37, 0.40) |
| 63 | Xipes0214 | 3 (3, 2) | 0.95 (0.95, 0.95) | 0.49 (0.53, 0.31) | 0.01 (0.00, 0.02) | 0.38 (0.47, 0.26) |
| 64 | Xipes0218 | 4 (4, 4) | 0.90 (0.82, 0.97) | 0.45 (0.51, 0.39) | 0.00 (0.00, 0.00) | 0.42 (0.55, 0.36) |
| 65 | Xipes0219 | 5 (4, 5) | 0.99 (0.99, 0.99) | 0.58 (0.36, 0.61) | 0.03 (0.01, 0.06) | 0.50 (0.32, 0.55) |
| 66 | Xipes0220 | 9 (8, 7) | 0.87 (0.88, 0.86) | 0.77 (0.60, 0.77) | 0.03 (0.01, 0.04) | 0.73 (0.55, 0.74) |
| 67 | Xipes0221 | 3 (3, 3) | 0.76 (0.72, 0.80) | 0.51 (0.49, 0.51) | 0.00 (0.00, 0.00) | 0.39 (0.38, 0.39) |
| 68 | Xipes0223 | 5 (5, 5) | 0.97 (0.96, 0.98) | 0.68 (0.65, 0.70) | 0.02 (0.02, 0.03) | 0.62 (0.58, 0.65) |
| 69 | Xipes0225 | 3 (3, 3) | 0.78 (0.72, 0.84) | 0.39 (0.41, 0.37) | 0.03 (0.02, 0.03) | 0.32 (0.34, 0.31) |
| 70 | Xipes0226 | 5 (4, 5) | 0.92 (0.89, 0.94) | 0.63 (0.59, 0.63) | 0.12 (0.10, 0.13) | 0.58 (0.54, 0.57) |
| 71 | Xipes0227 | 3 (3, 3) | 0.99 (0.98, 0.99) | 0.29 (0.30, 0.29) | 0.01 (0.00, 0.03) | 0.27 (0.27, 0.27) |
| 72 | Xipes0233 | 21 (13, 21) | 0.98 (0.98, 0.98) | 0.85 (0.67, 0.91) | 0.06 (0.03, 0.08) | 0.84 (0.64, 0.90) |
| 73 | Xpsmp2040 | 6 (4, 5) | 0.80 (0.83, 0.78) | 0.64 (0.53, 0.68) | 0.00 (0.00, 0.00) | 0.57 (0.47, 0.62) |
| 74 | Xpsmp2070 | 28 (19, 23) | 0.86 (0.88, 0.84) | 0.90 (0.80, 0.92) | 0.01 (0.01, 0.01) | 0.90 (0.78, 0.91) |
| 75 | Xpsmp2077 | 10 (8, 8) | 0.97 (0.99, 0.95) | 0.65 (0.67, 0.53) | 0.04 (0.04, 0.04) | 0.61 (0.61, 0.50) |
| 76 | Xpsmp2201 | 6 (6, 6) | 0.93 (0.93, 0.93) | 0.67 (0.54, 0.75) | 0.02 (0.01, 0.03) | 0.64 (0.51, 0.72) |
| 77 | Xpsmp2203 | 8 (7, 8) | 0.76 (0.76, 0.77) | 0.76 (0.58, 0.83) | 0.00 (0.00, 0.01) | 0.73 (0.54, 0.80) |
| 78 | Xpsmp2204 | 3 (2, 3) | 0.89 (0.85, 0.93) | 0.18 (0.03, 0.28) | 0.02 (0.00, 0.03) | 0.16 (0.03, 0.25) |
| 79 | Xpsmp2206 | 8 (7, 7) | 0.90 (0.88, 0.91) | 0.81 (0.76, 0.83) | 0.00 (0.00, 0.00) | 0.79 (0.73, 0.81) |
| 80 | Xpsmp2208 | 8 (6, 5) | 0.80 (0.87, 0.74) | 0.41 (0.16, 0.60) | 0.04 (0.01, 0.07) | 0.39 (0.16, 0.56) |
| 81 | Xpsmp2214 | 3 (3, 3) | 0.79 (0.79, 0.79) | 0.62 (0.54, 0.66) | 0.04 (0.04, 0.05) | 0.55 (0.48, 0.59) |
| 82 | Xpsmp2215 | 3 (2, 3) | 0.95 (0.96, 0.94) | 0.36 (0.48, 0.11) | 0.00 (0.00, 0.00) | 0.30 (0.37, 0.11) |
| 83 | Xpsmp2227 | 7 (5, 6) | 0.87 (0.84, 0.90) | 0.53 (0.57, 0.46) | 0.03 (0.01, 0.04) | 0.44 (0.47, 0.40) |
| 84 | Xpsmp2235 | 2 (2, 2) | 0.98 (0.99, 0.97) | 0.06 (0.05, 0.07) | 0.00 (0.00, 0.00) | 0.06 (0.05, 0.06) |
| 85 | Xpsmp2236 | 3 (3, 3) | 0.82 (0.79, 0.85) | 0.27 (0.18, 0.33) | 0.00 (0.00, 0.01) | 0.24 (0.17, 0.30) |
| 86 | Xpsmp2237 | 8 (5, 7) | 0.74 (0.78, 0.71) | 0.46 (0.35, 0.55) | 0.03 (0.03, 0.03) | 0.44 (0.33, 0.51) |
| 87 | Xpsmp2240 | 3 (3, 3) | 0.90 (0.88, 0.92) | 0.27 (0.44, 0.05) | 0.00 (0.00, 0.00) | 0.24 (0.35, 0.05) |
| 88 | Xpsmp2253 | 2 (2, 2) | 0.88 (0.84, 0.92) | 0.08 (0.12, 0.04) | 0.00 (0.00, 0.00) | 0.07 (0.11, 0.03) |
|  | **Total** | **532 (443, 476)** | **78.38** | **48.01** | **2.03** | **43.88** |
|  | **Mean** | **6.05 (5.03, 5.41)** | **0.89 (0.89, 0.89)** | **0.55 (0.49, 0.55)** | **0.02 (0.01, 0.03)** | **0.50 (0.44, 0.50)** |

Note: Allele number, availability, gene diversity, heterozygosity and PIC for the B- and R- lines are given in parenthesis, respectively.
